# Solving the Puzzle of Unusual Excited-State Proton Transfer in 2,5-Bis(6-methyl-2-benzoxazolyl)phenol

**DOI:** 10.1021/acs.jpca.1c10030

**Published:** 2022-03-14

**Authors:** Jacek Dobkowski, Michał Kijak, Sylwester Gawinkowski, Elena Karpiuk, Mariusz Pietrzak, Igor V. Sazanovich, Jacek Waluk

**Affiliations:** † Institute of Physical Chemistry, Polish Academy of Science, Kasprzaka 44/52, 01-224 Warsaw, Poland; ‡ Faculty of Mathematics and Science, Cardinal Stefan Wyszyński University, Dewajtis 5, 01-815 Warsaw, Poland

## Abstract

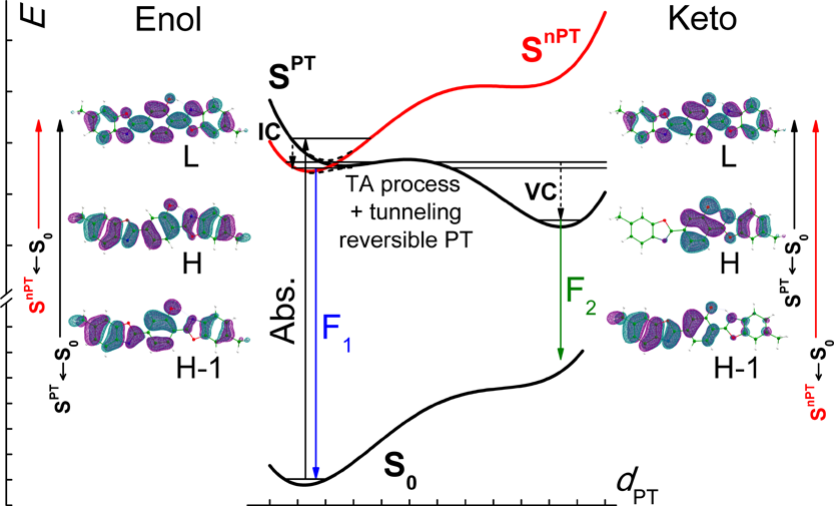

2,5-Bis(6-methyl-2-benzoxazolyl)phenol (**BMP**) exhibits an ultrafast excited-state intramolecular proton transfer (ESIPT) when isolated in supersonic jets, whereas in condensed phases the phototautomerization is orders of magnitude slower. This unusual situation leads to nontypical photophysical characteristics: dual fluorescence is observed for **BMP** in solution, whereas only a single emission, originating from the phototautomer, is detected for the ultracold isolated molecules. In order to understand the completely different behavior in the two regimes, detailed photophysical studies have been carried out. Kinetic and thermodynamic parameters of ESIPT were determined from stationary and transient picosecond absorption and emission for **BMP** in different solvents in a broad temperature range. These studies were combined with time-dependent- density functional theory quantum-chemical modeling. The excited-state double-well potential for **BMP** and its methyl-free analogue were calculated by applying different hybrid functionals and compared with the results obtained for another proton-transferring molecule, 2,5-bis(5-ethyl-2-benzoxazolyl)hydroquinone (**DE-BBHQ**). The results lead to the model that explains the difference in proton-transfer properties of **BMP** in vacuum and in the condensed phase by inversion of the two lowest singlet states occurring along the PT coordinate.

## Introduction

Absorption of a photon can initiate numerous intramolecular processes. Among these, the excited-state intramolecular proton transfer (ESIPT) reaction plays a prominent role.^1−32^ ESIPT occurs in molecules that have proton-donating and proton-accepting centers electronically conjugated through the molecular skeleton and which, additionally, show significant changes in their electron density distribution after excitation.^17,24^

**Scheme 1:**
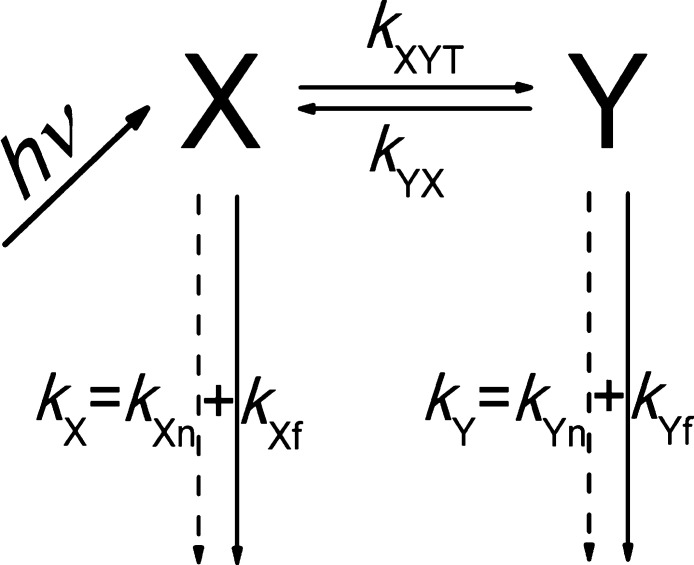
Diagram of the ESIPT Process

The kinetics of the ESIPT reaction is described formally by Scheme 1, where *X* and *Y* represent the primarily excited species and the product of the reaction, frequently the enol and keto forms; *k*_*X*f/*Y*f_ and *k*_*X*n/*Y*n_ denote their radiative/nonradiative rate constants; and *k*_*X*_ and *k*_*Y*_ are the results of summation: *k*_*X*f_ + *k*_*X*n_ and *k*_*Y*f_ + *k*_*Y*n_, respectively; *k*_*XYT*_ and *k*_*YX*_ are forward and backward PT rates; “*T*” indicates that temperature-independent tunneling was taken into account.

This simple scheme of single PT can be a drastic oversimplification. The ESIPT reaction is frequently a complicated multidimensional process described in terms of quantum mechanics as delocalization of the proton wave function over the regions of primarily excited (*X*) and secondary (*Y*) species occupying two minima on the energy hypersurface.^14,25,33,34^ Since the proton wave function is more localized than that of the electron, it is reasonable to postulate that coupling between primary and secondary species is very sensitive to the distance between proton-donating and proton-accepting nuclei.^35^ This distance can be considerably modulated by some vibrations.^19,35−37^

**Scheme 2:**
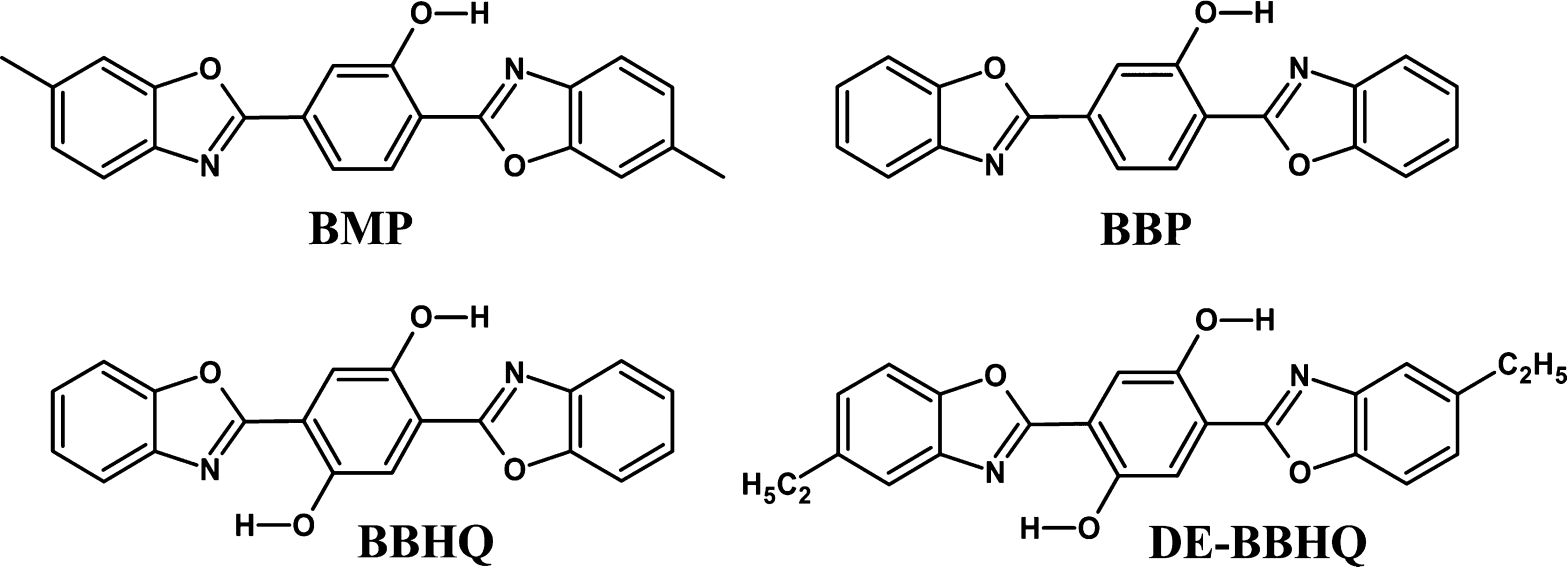
Formulas of **BMP** and Related Species

The subject of our study is 2,5-bis(6-methyl-2-benzoxazolyl)phenol (**BMP**), a member of the bis-benzoxazoles group (Scheme 2). These molecules often exhibit dual or even triple emission due to the single or double PT occurring in the electronically excited state. The description of the photoreaction mechanism is quite complex since it must include several factors: the role of tunneling, possibility of reverse tautomerization, cooperativity between two proton-transferring centers, and even rotameric equilibria.^35,36,38−42^

**Scheme 3:**
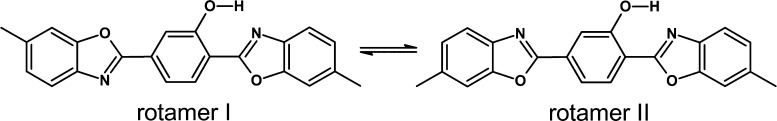
Rotamers of **BMP**

Isolated **BMP** has been intensely investigated using the supersonic jet techniques.^35,37^ Interestingly, under these conditions, a “normal”, short-wavelength fluorescence, expected to occur from the initially excited species, was not detected. Consequently, the laser-induced fluorescence excitation (LIF) spectrum was recorded only upon observation of the “red” fluorescence. The most fundamental difference between the LIF spectrum of a nondeuterated molecule and deuterated molecule is a significant change in the full width at half-maximum (FWHM) of lines. For the (0,0) transition, it is reduced from 74 to 6.6 ± 0.2 cm^–1^. The upper limits of the proton (nondeuterated molecule) or deuteron (deuterated molecule) transfer rate constants have been estimated using the formula (FWHM) = (2π*c*τ)^−1^, where τ is the excited-state lifetime and *c* is the velocity of light, to be *k*_*XY*_ = 1.4 × 10^13^/1.24 × 10^12^ s^–1^, respectively.^35,37^ Replacement of the hydrogen atom with deuterium reduces the ESIPT rate constant approximately by a factor of 10. The results of the hole burning experiments indicate that two different forms coexist in the case of the *d*_1_ (OD) isotopomer of **BMP** in the ground state. These two species were ascribed to rotamers generated by the rotation of the “free,” non-hydrogen-bonded benzoxazolyl group (see Scheme 3).^35,37^ Rotamer II has the (0,0) transition shifted by 115 cm^–1^ to blue with respect to the origin band of rotamer I. It should be mentioned that for bis-benzoxazoles with two OH groups, for example, **BBHQ**, the presence of only one form is expected and observed. For this class of bis-benzoxazoles, single and double PT reactions have been considered.^40^ The possibility of two consecutive PTs in **DE-BBHQ/BBHQ** was predicted by quantum-chemical modeling,^34,40^ and recently, a third fluorescence band was observed for these systems in the infrared region.^42^

**Scheme 4:**
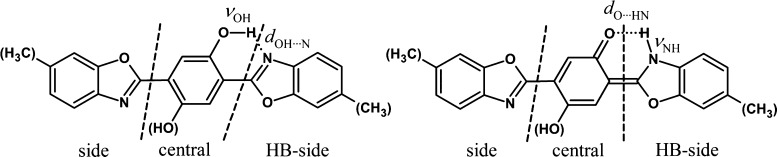
Dashed Lines Indicate Borders of **BMP** Subgroups for Which Δ*q* Values Were Calculated (See Table 4 for Details)

Contrary to the case of jet-isolated **BMP**, its solutions exhibit dual fluorescence. This unusual behavior prompted us to study the origin of this difference. The goal of this work is to compare the excited-state energy dissipation processes associated with ESIPT reaction of **BMP** occurring in vacuum and in the condensed phase. To understand the nature of the states involved in ESIPT, quantum-chemical modeling was performed. A combination of the experimental and theoretical findings leads to a model that postulates solvent-induced energy inversion of the two lowest excited states in **BMP**.

## Experimental and Computational Details

**BMP** was synthesized as described previously.^43^ 2-Methyltetrahydrofuran (MTHF, Merck for synthesis) was repeatedly distilled over CaCl_2_. Butyronitrile (BuCN, Merck for synthesis) was repeatedly distilled over CaCl_2_ and P_2_O_5_. 3-Methylpentane (3MP) and *n*-hexane (Merck, spectral grade) were used without purification. NMR spectra were obtained using a Bruker AVANCE II 300 spectrometer operating at 300.17 MHz for ^1^H. Stationary absorption spectra were recorded with a Shimadzu UV 3100 spectrometer. Stationary fluorescence spectra were measured using the Jasny^44^ or the FS900 Edinburgh Instrument spectrofluorimeters equipped with an Oxford cryostat or closed-cycle helium cryostat (Advanced Research Systems Inc.). The spectra were corrected for the instrumental response using fluorescence standards. Fluorescence quantum yields were determined using quinine sulfate in 0.05 M H_2_SO_4_ as a standard (φ = 0.51).^45^ Fluorescence decays in the nanosecond domain were recorded with the single-photon counting unit (Edinburgh Instrument); λ_exc_ = 375 nm. The temporal resolution is 0.1 ns. For recording the transient absorption (TA) spectra, a homebuilt picosecond spectrometer was used. Briefly, pulses of 1.5 ps duration (1055 nm) and an energy of 4 mJ with a repetition of 33 Hz are provided by a Light Conversion (Vilnius, Lithuania) Nd:glass laser, λ_exc_ = 351.7 nm (third harmonic of the Nd:glass laser). The temporal resolution of the spectrometer is 2.5 ps. The time-resolved fluorescence (TRF) spectra were recorded by means of a homemade picosecond spectrofluorimeter described in detail elsewhere.^46^ In short, the first beam (352 nm) is used for excitation. The second beam passes through an optical Kerr shutter and opens it. The fluorescence can be transmitted by the shutter only for the time period in which the opening pulse penetrates the Kerr medium. The opening pulse is delayed with respect to the excitation by an optical delay line (a maximum delay of 3000 ps, 0.1 ps/step). The delay time is calculated with respect to the maximum of the excitation pulse. The fluorescence is transmitted to the detection system by a quartz fiber. The detection system consists of a polychromator (Acton SpectraPro-275) and a CCD detector (Princeton Instruments, Inc.). The temporal resolution of the spectrofluorimeter is 6.5 ps. The spectra were corrected for the instrumental response.

Quantum-chemical modeling of the studied systems was performed using density functional theory (DFT) and its time-dependent formalism (TD-DFT) for the ground state and excited states, respectively. The hybrid B3LYP functional and 6-31+G(d,p) basis set were used. For the excited states, we also checked two range-separated functionals: a long-range corrected CAM-B3LYP and meta-GGA highly parameterized Minnesota M11. The unrestricted DFT formalism was used to describe the lowest triplet state. For modeling of our system in a solvent environment, the polarized continuum method with the integral equation formalism (IEFPCM) and with the self-consistent approach for the excited-state energies was chosen. Construction of the PT path was achieved via fixing of the O–H or N–H distances (on the keto and enol forms, respectively) during optimization. Transition states (TSs) were fully optimized by the TS option. The character of all the obtained stationary points was confirmed by frequency analysis. The electrostatic-potential-fitted atomic charges have been obtained according to the CHelpG scheme. The Gaussian 09 suite of programs was used.^47^

## Results

### Room- and Low-Temperature NMR, Stationary Absorption, and Fluorescence

To determine the value of the ground-state barrier for the rotation of the free benzoxazolyl group, ^1^H NMR spectra of **BMP** were recorded as a function of temperature down to 173 K in deuterated tetrahydrofuran (THF) (Figure S1). No splitting or broadening of the NMR lines associated with H6-singlet at 7.92 ppm (294 K) and H4-doublet at 7.91 ppm (294 K) was observed, which indicates that either two **BMP** rotamers are in a fast exchange regime or there exists only one rotamer.

**Figure 1:**
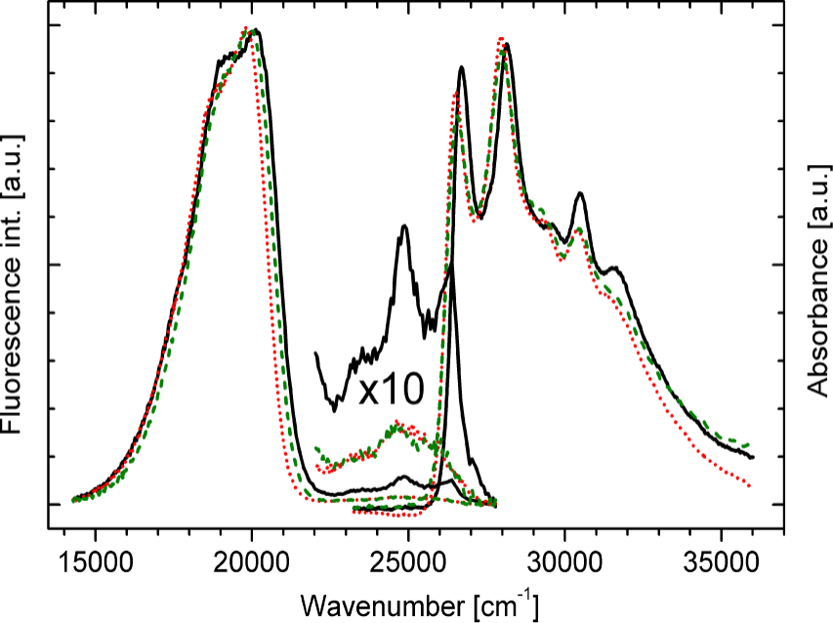
Room-temperature absorption and fluorescence spectra of **BMP** recorded in 3MP (black solid line), MTHF (red dotted line), and BuCN (green dashed line); λ_exc_ = 355 nm.

Room-temperature absorption and fluorescence spectra of **BMP** were recorded in 3MP (nonpolar solvent), MTHF, and BuCN, characterized by dielectric constants of 1.9, 7.5, and 20.3, respectively (Figure 1). The absorption spectra show a well-defined structure with maxima at 26 700, 28 200, 29 600, 30 500, and 31 600 cm^–1^.

Independent of the solvent polarity, electronic excitation of **BMP** results in dual fluorescence (Figure 1). The main, low-energy fluorescence band with a maximum at about 20 000 cm^–1^ exhibits a large Stokes shift (around 7000 cm^–1^). Contrary to this, a high-energy fluorescence shows a typical Stokes shift. This emission at room temperature exhibits a vibrational structure only in a nonpolar environment (26 400, 24 900, 23 500 cm^–1^). The fluorescence excitation spectra of **BMP** recorded by monitoring high- and low-energy fluorescence bands are in good agreement with the absorption spectrum.^35^ Excitation wavelength dependence of the **BMP** emission was not observed.

The emission and absorption spectra of **BMP** in 3MP recorded at low temperatures are presented in Figure S2. A concentration-dependent change of absorption and fluorescence spectra is observed below 153 K. The structure of the absorption spectrum disappears. Simultaneously, in the emission spectrum, a new band arises at about 22 000 cm^–1^. These experimental results indicate that in nonpolar solvents at low temperatures, ground-state aggregation takes place.

**Figure 2:**
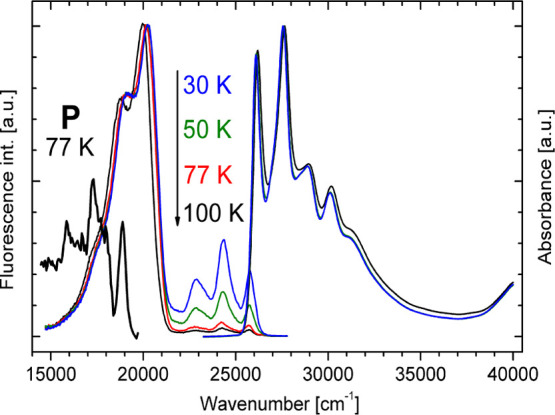
Low-temperature normalized absorption, fluorescence, and phosphorescence (*P*) spectra of **BMP** in MTHF recorded at *T* = 100 K (black), 77 K (red), 50 K (green), and 30 K (blue). The phosphorescence was normalized to 0.5.

Low-temperature spectra of **BMP** recorded in MTHF are shown in Figure 2. The spectral position and vibrational pattern of the absorption spectrum of **BMP** in MTHF do not change with a temperature below 100 K. For temperatures higher than 100 K, a blue shift of the first absorption band is observed. This temperature-dependent transformation of the spectrum can be associated with temperature-dependent populations of the rotamers in the ground state. The vibrational structure of the high-energy fluorescence appears at temperatures lower than 223 K. In rigid MTHF, a structured phosphorescence is also observed, with the (0,0) transition at 18 850 cm^–1^.

For the temperature range of 163–294 K, the fluorescence spectrum of **BMP** in BuCN (ε = 20.3) undergoes a similar transformation as in the case of MTHF.

### Fluorescence Quantum Yield of **BMP** as a Function of Temperature

The room-temperature total fluorescence quantum yields (φ_*T*_) of **BMP** in *n*-hexane, MTHF, and BuCN are 0.27, 0.28, and 0.25, respectively. The quantum yield of the blue fluorescence (φ_*X*_) is 0.017 in *n*-hexane, 0.005 in MTHF, and 0.004 in BuCN (estimated error ± 15%).

**Figure 3:**
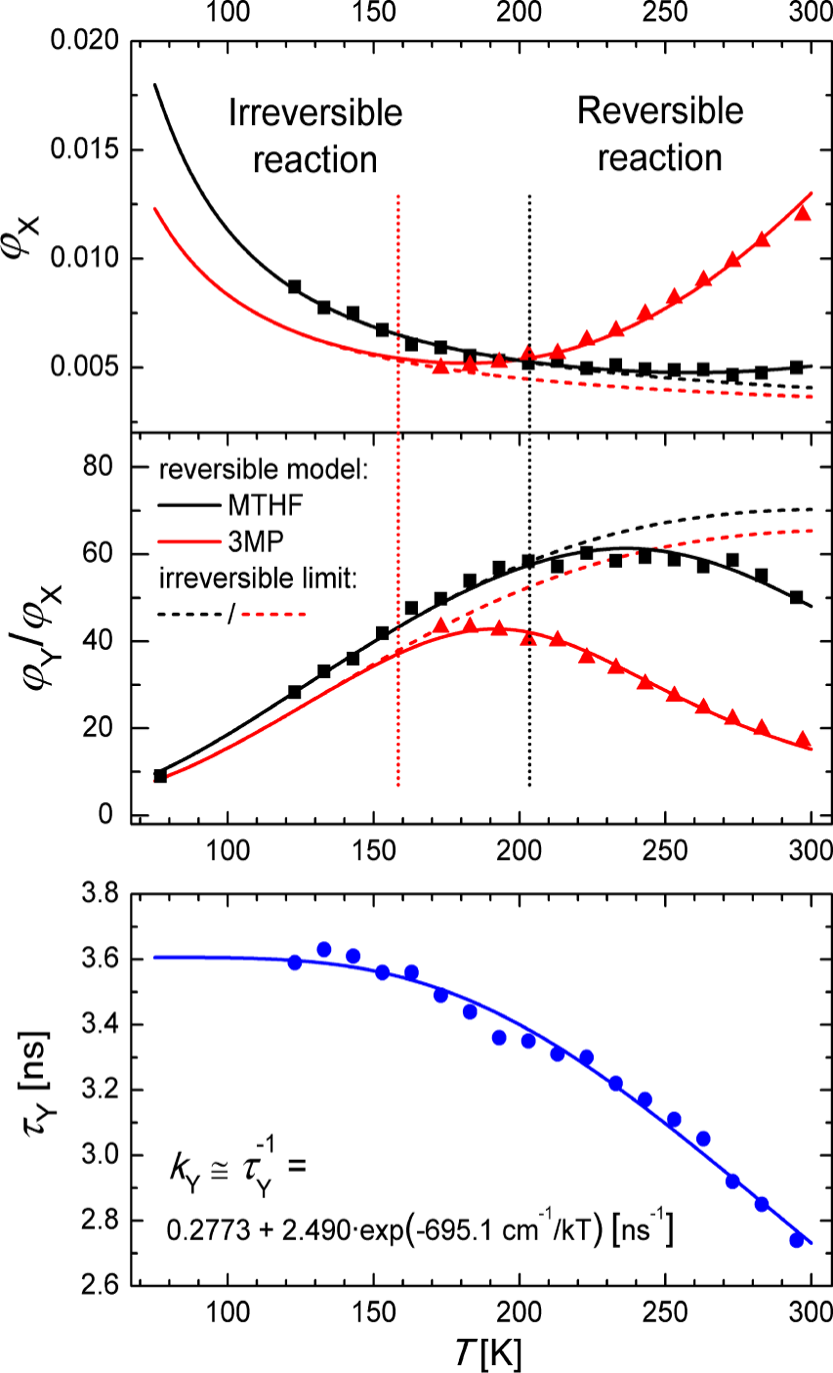
Top: quantum yield of the high-energy fluorescence (φ_*X*_) and the low to high energy fluorescence quantum yield ratio (φ_*Y*_/φ_*X*_) for **BMP** in 3MP (red triangles) and MTHF (black squares) recorded in the temperature ranges of 172–297 and 77–295 K, respectively. Solid lines indicate the results of fitting with formulae 1 and 3, respectively (fitted parameters given in Table 1). Dashed lines indicate the irreversible limit of the reaction. Bottom: temperature dependence of the low-energy fluorescence lifetime (circles) of **BMP** in MTHF with the result of exponential fitting (solid line, equation).

**Figure 4:**
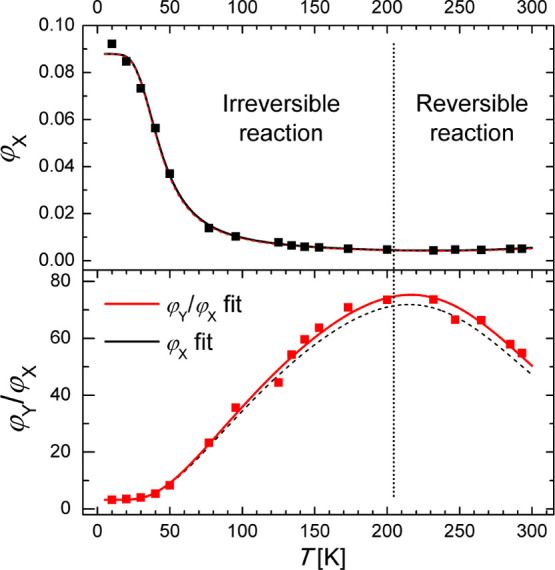
Quantum yield of the high-energy fluorescence (φ_*X*_) and the low to high energy fluorescence quantum yield ratio (φ_*Y*_/φ_*X*_) for **BMP** in MTHF recorded in the temperature range of 10–293 K, with detailed description in the text. Solid lines indicate the results of fitting of φ_*X*_(*T*) and φ_*Y*_(*T*)/φ_*X*_(*T*) data sets with eqs 1 and 3, respectively (fitted parameters given in Table 1). Dashed lines represent the simulated behavior of φ_*X*_(*T*) and φ_*Y*_(*T*)/φ_*X*_(*T*) calculated using the parameters obtained from fitting of φ_*Y*_(*T*)/φ_*X*_(*T*) and φ_*X*_(*T*) data sets, respectively.

The fluorescence spectra of **BMP** were measured as a function of temperature in 3MP (for the 173–297 K range), MTHF (77, 123–295 K), and BuCN (163–294 K) and in the case of MTHF additionally within the 10–293 K range. The quantum yields for **BMP** in 3MP are reported only in the temperature region where fluorescence can be safely assigned to the emission of the **BMP** monomer. The quantum yields of the primary (φ_*X*_) and secondary (φ_*Y*_) emissions and the low to high energy fluorescence quantum yield ratio (φ_*Y*_/φ_*X*_) are presented in Figures 3, 4, S3, and S4. The lifetime of the red fluorescence (τ_*Y*_) of **BMP** in MTHF was measured in the temperature range of 123–295 K (Figure 3, bottom). A simple analysis of the plot of ln(φ_*X*_) versus 1/*T*^48^ for **BMP** in MTHF indicates that the values of the barriers for the forward and backward processes lie in the ranges of 90–140 and 1500–1900 cm^–1^, respectively. Thus, even at room temperature, the forward reaction is almost 3 orders of magnitude faster than the backward one. Therefore, an approximation of τ_*Y*_(*T*)^−1^ ≅ *k*_*Y*_(*T*) is well justified and was used. The Arrhenius type behavior of the temperature-dependent term in *k*_*Y*_ was assumed to simulate *k*_*Y*_(*T*) and extrapolate it below 123 K. The *k*_*Y*f_ value of (9.5 ± 2.0) × 10^7^ s^–1^ was calculated as φ_*Y*_/τ_*Y*_ at temperatures corresponding to the irreversible reaction range.

The quantum yield of the red fluorescence of **BMP** (φ_*Y*_) measured as a function of the temperature in solvents of different polarities behaves similarly and reaches the maximum at 200–230 K (Figure S4). Contrary to this, the shape of the φ_*X*_(*T*) function depends on the solvent polarity (Figures 3, S3). In polar solvents (MTHF, BuCN), φ_*X*_(*T*) forms a plateau in the range of 200–295 K and increases with the decrease of the temperature below 200 K. In nonpolar 3MP, φ_*X*_ decreases upon cooling in the whole accessible temperature range of 173–297 K.

The φ_*X*_(*T*) and φ_*Y*_(*T*)/φ_*X*_(*T*) data sets for **BMP** in 3MP (Figure 3) and MTHF (for two temperature ranges, Figures 3 and 4) were fitted with formulae 1 and 3, respectively (Table 1). The data sets for **BMP** in MTHF in a wider temperature range of 10–293 K were obtained from separate measurements in two nonoverlapping temperature ranges: 125–293 (A) and 10–95 K (B). Due to this, the fitting procedure of the φ_*X*_(*T*) data set was initially performed for range A only (with the value of φ_*X*_(293 K) known), and then, the value of φ_*X*_ extrapolated to 100 K was taken as a reference point to obtain the quantum yield values for range B. Such corrected data are presented in Figure 4, whereas the raw data set (φ′_*X*_(*T*)) is presented in Figure S5. The fitted values of *E*_*XY*_, *E*_*YX*_, and *A*_*XY*_ obtained for φ_*X*_(*T*) and φ′_*X*_(*T*) data sets are similar (see Table 1 and caption to Figure S5), whereas the value of *k*_*X*_ differs significantly. The fluorescence quantum yields of both bands of **BMP** in BuCN (ε = 20.3) in the whole temperature range of 163–294 K change similarly as in the case of less polar MTHF (Figures S3, S4).

**Table 1:** Kinetic Parameters of **BMP** in MTHF and 3MP Determined from the Fitting of φ_*X*_(*T*) and φ_*Y*_(*T*)/φ_*X*_(*T*) Data Sets with formulae 1 and 3, Respectively, in Different Temperature Ranges (as in Figures 3 and 4)^a^

	MTHF 10–293 K φ_*Y*_/φ_*X*_(*T*)	MTHF 10–293 K φ_*X*_(*T*)	MTHF 77–295 K φ_*Y*_/φ_*X*_(*T*)	MTHF 123–295 K φ_*X*_(*T*)	3MP 173–297 K φ_*Y*_/φ_*X*_(*T*)	3MP 173–297 K φ_*X*_(*T*)
*E*_*XY*_ [cm^–1^]	119 ± 5	119 ± 3	184 ± 7	116 ± 6	199 ± 3	92 ± 3
*E*_*YX*_ [cm^–1^]	1550 ± 30	1530 ± 50	1640 ± 20	1680 ± 40	1230 ± 10	1190 ± 10
*A*_*XY*_ [10^9^ s^–1^]	370 ± 20	350 ± 30	440 ± 30	290 ± 20	440^b^	290^b^
*k*_*T*_ [10^9^ s^–1^]	6.5 ± 0.8	6.5^b^	6.5^b^	6.5^b^	6.5^b^	6.5^b^
*k*_*X*_ [10^9^ s^–1^]		1.5 ± 0.2		1.5^b^		1.5^b^

a *k*_*X*f_ = (7 ± 2) × 10^8^ s^–1^, *k*_*Y*f_ = (9.5 ± 2.0) × 10^7^ s^–1^, and *k*_*Y*_(*T*) = τ_*Y*_(*T*)^−1^ (Figure 3, bottom) are evaluated for **BMP** in MTHF.

b Parameter taken from a different fit and fixed.

### Modeling of the ESIPT Kinetics

In the case of the excited-state reaction described by general Scheme 1, the quantum yields of the primary (φ_*X*_) and secondary (φ_*Y*_) fluorescences as well as the φ_*Y*_/φ_*X*_ ratio measured as a function of temperature can be described by the following equations^38,39,48^
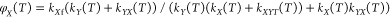
1
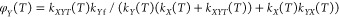
2
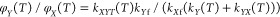
3where *k*_*XYT*_(*T*) = *k*_*T*_ + *k*_*XY*_(*T*) and *k*_*T*_ accounts for possible temperature-independent tunneling.

Assuming the Arrhenius dependence of forward and backward PT rates, *k*_*XY*/*YX*_(*T*) = *A*_*XY*/*YX*_ exp(−*E*_*XY*/*YX*_/*kT*), where *E*_*XY*/*YX*_ is the forward/backward ESIPT reaction energy barrier and *k* is the Boltzmann constant, and neglecting temperature dependence of *k*_*X*_ (*k*_*X*_(*T*) = *k*_*X*_) leads to algebraic expressions with nine independent parameters (eight constants: *k*_*X*f_, *k*_*Y*f_, *k*_*X*_, *k*_*T*_, *A*_*XY*_, *E*_*XY*_, *A*_*YX*_, *E*_*YX*_, and *k*_*Y*_(*T*) as a known function, see Figure 3). Three of these, *k*_*X*f_, *k*_*Y*f_, and *k*_*Y*_(*T*), were determined experimentally, where *k*_*X*f_ = φ_*X*_/τ_*X*_ due to the limited temporal resolution of the apparatus was established at 93 K only and was treated as temperature-independent value, whereas *k*_*Y*f_ = φ_*Y*_/τ_*Y*_ was measured in the temperature region of 125–294 K. Some drift of the *k*_*Y*f_ value was observed below 173 K. The value of *k*_*Y*f_ = 9.5 × 10^7^ s^–1^ was determined at 193 K, where the contribution of the reverse reaction can be neglected. An additional assumption that *A*_*XY*_ = *A*_*YX*_ reduces the number of unknown parameters to five. Moreover, in the φ_*Y*_(*T*)/φ_*X*_(*T*) ratio (eq 3), *k*_*X*_ is not present. Additionally, from an experimental point of view, determination of the ratio is free of some errors inherent to the quantum yield determination. Having this in mind, we paid more attention to the φ_*Y*_(*T*)/φ_*X*_(*T*) fitting. To check the reliability of our approach, independent fits of φ_*X*_(*T*) data sets were also performed.

Some additional remarks had to be made. It turned out that *k*_*T*_ is significant (comparable with *k*_*XY*_(*T*)) only at temperatures lower than 80 K. Consequently, the *k*_*T*_ value can be reliably determined only from fits for **BMP** in MTHF in the low-temperature range (Figure 4). Moreover, upon fitting of φ_*X*_(*T*) with eq 1, it was not possible to obtain *k*_*T*_ and *k*_*X*_ independently (in the dominant term, they occur as a sum). Therefore, the *k*_*T*_ value was taken from the φ_*Y*_(*T*)/φ_*X*_(*T*) fit and fixed. For narrower temperature ranges (Figure 3), even with *k*_*T*_ fixed, we failed to estimate *k*_*X*_ reliably, and in the case of 3MP, also the *A*_*XY*_ value. It can be explained by a high degree of dependency between *k*_*X*_ and *A*_*XY*_ in that temperature range and the limited number of experimental points. Due to this, some parameters had to be taken from different fits and fixed, as is indicated in Table 1.

It should be pointed out that taking into account substantial errors in the estimation of quantum yields, lifetimes, and parameters derived from them (*k*_*X*f_, *k*_*Y*f_, *k*_*Y*_(*T*)) does not change the fitted reaction barriers (*E*_*XY*_ and *E*_*YX*_) significantly (less than 10%), in contrast to *A*_*XY*_, *k*_*X*_, and *k*_*T*_ values. Moreover, the parameters determined from the fitting of the experimental data sets obtained for the temperature range of 10–294 K seem to be more credible than those obtained from the limited temperature range.

### Time-Resolved Experiments in the Picosecond Time Domain

The room-temperature decay curve evaluated for the blue band of TRF spectra of **BMP** exhibits a biexponential pattern, suggesting that the ESIPT reaction is reversible.^48^ Due to the temporal resolution (6.5 ps, of the order of the short component of the decay) and the limited time window of TRF spectra registration (of the order of the long component), a lifetime fitting procedure was not performed. The amplitude of the fast component was about 3 times higher than the amplitude of the long component.

**Figure 5:**
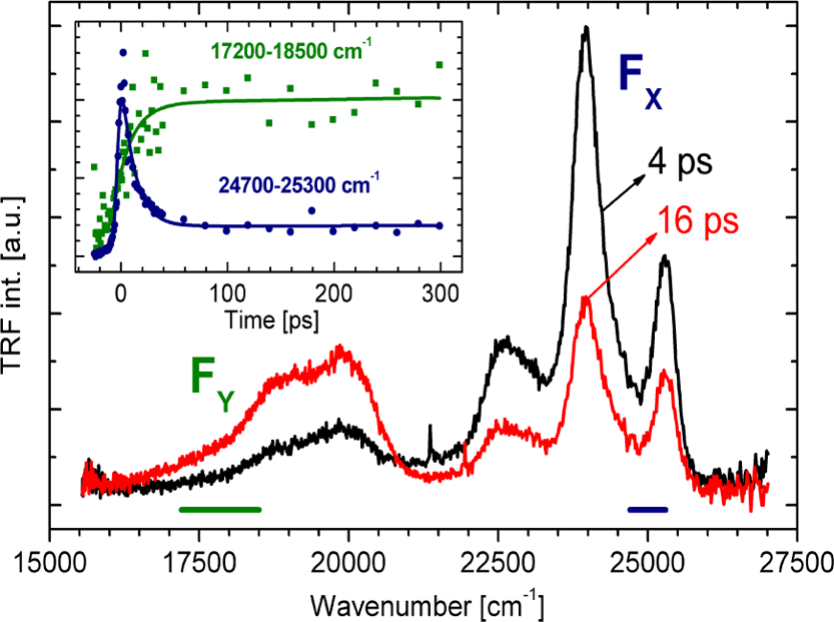
TRF spectra of **BMP** in MTHF at *T* = 93 K recorded for selected delay times. The inset shows the time evolution of the primary (*F*_*X*_, blue circles) and secondary (*F*_*Y*_, green squares) fluorescence with the results of fitting (solid lines): *F*_*X*_ (τ_1_ = 15 ± 3 ps, τ_2_ > 1000 ps) and *F*_*Y*_ (^R^τ_1_ = 17 ± 4 ps, τ_2_ > 1000 ps, R—rise).

Low-temperature TRF spectra of **BMP** in MTHF recorded at 93 K consist of a structured high-energy band and a broad low-energy emission (Figure 5). The decay of the blue emission is accompanied by a simultaneous rise of the secondary TRF band. The decay and rise times are 15 ± 3 and 17 ± 4 ps, respectively. The long component of the decay curve is associated with the leaking of the Kerr shutter and should be treated, in this time window, as constant.

**Figure 6:**
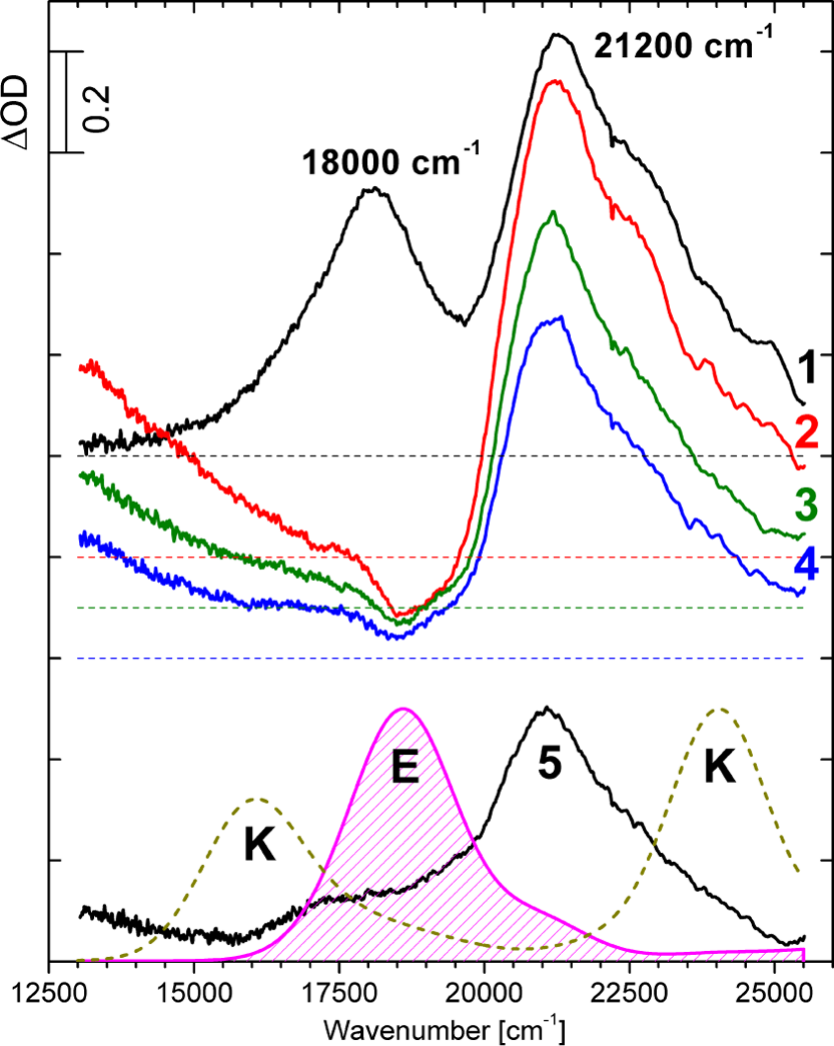
Room-temperature TA spectra of **BMP** in MTHF recorded for selected delay times: 3 (1), 9 (2), 1000 (3), 1600 (4), and 2800 ps (5); in the case of (1–4), an offset was applied for better visualization. TD-UB3LYP-calculated normalized TA spectra of the triplet state of the enol (E) and keto (K) forms of **BMP**.

Room-temperature TA spectra of **BMP** in MTHF are presented in Figure 6. Just after excitation, two prevailing bands with the maxima at 18 000 and 21 200 cm^–1^ are observed. The decay time of the first band is comparable with the temporal resolution of the apparatus, whereas that of the second band is in the μs domain.^37^ It is reasonable to assign this long-lived TA band to T_*n*_ ← T_1_ absorption. T_*n*_ ← T_1_ transitions were calculated for the enol and keto forms of **BMP** (Figure 6, bottom). It should be mentioned that the decrease of the intensity of the high-energy TA band is observed in the ns time domain, which can suggest that the contribution of S_*n*_ ← S_1_ absorption of the secondary form cannot be neglected in the spectral region of 21 000–25 000 cm^–1^.

**Figure 7:**
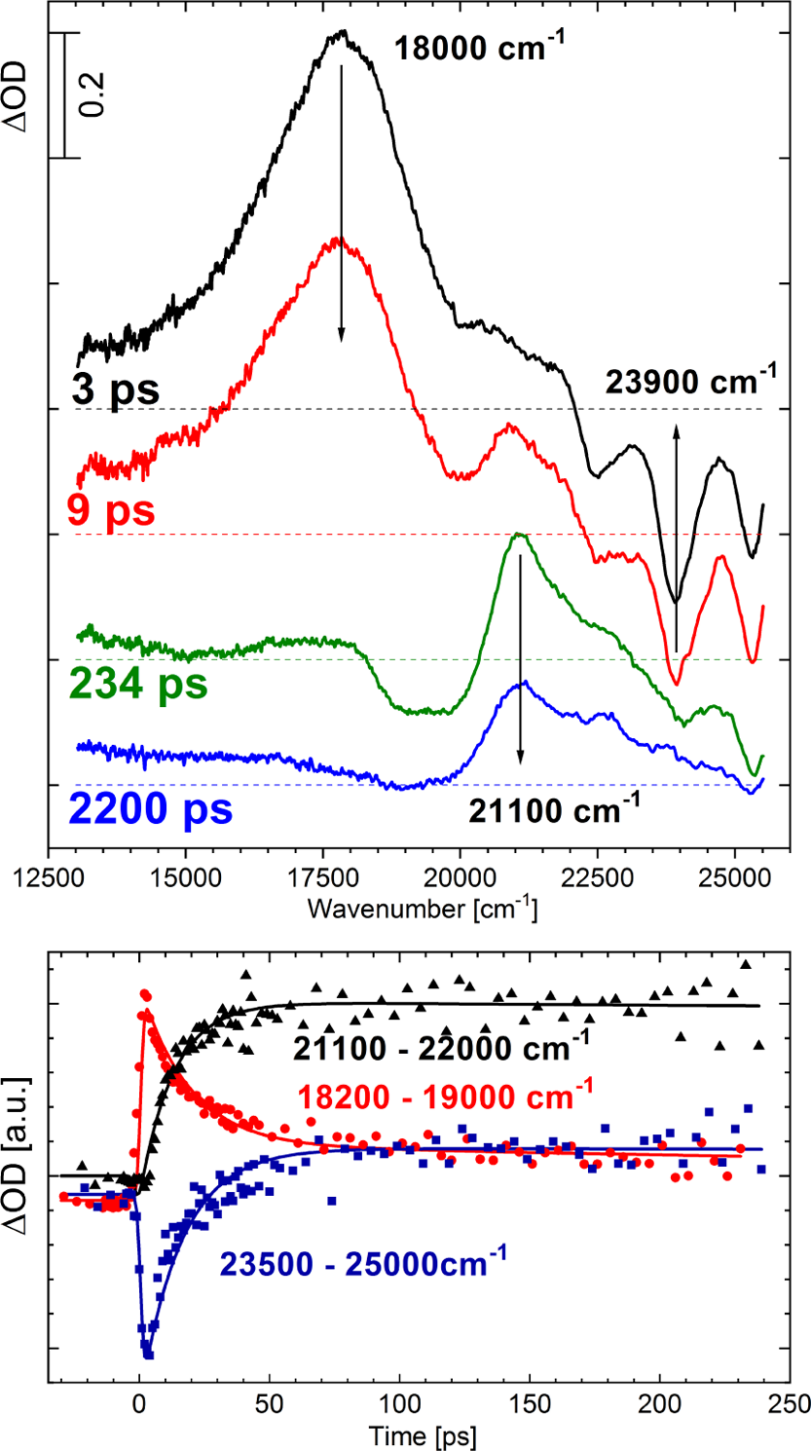
Top: TA spectra of **BMP** in MTHF recorded at 93 K as a function of the delay time; an offset was applied for better visualization. Bottom: normalized kinetic traces of the SE at 23 900 cm^–1^ (squares; lines, biexponential fit: τ_1_ = 16 ± 3 ps, τ_2_ > 1000 ps) and TA bands with a maximum at 18 000 cm^–1^ (circles; τ_1_ = 19 ± 4 ps, τ_2_ > 1000 ps) and at 21 100 cm (triangles; τ_1_^R^ = 16 ± 4 ps, *t*_2_ > 1000 ps).

Low-temperature TA spectra of **BMP** in MTHF are presented in Figure 7. Structured stimulated emission (SE) is observed within the spectral region of 22 000–25 000 cm^–1^, resembling the inverted stationary fluorescence of the enol form. The lifetime evaluated from its decay is equal to τ_1_ = 16 ± 3 ps (Figure 7, bottom). The decay time of the TA band with a maximum at 18 000 cm^–1^ is 19 ± 4 ps. It indicates that this TA band corresponds to the S_*n*_ ← S_1_ transitions of the primary excited form. The rise time of the TA band with a maximum at 21 100 cm^–1^ is equal to 16 ± 4 ps. Having in mind the long decay of this TA band at room temperature (about 1.5 μs^37^) and equality of its rise time and the decay time of the SE of the enol form, this band can be assigned to the T_*n*_ ← T_1_ absorption of the primary form.

For **BMP** in MTHF at 93 K, the blue fluorescence quantum yield and decay time are φ_*X*_(93 K) = 0.011 ± 0.002 and τ(93 K) = 16 ± 3 ps, respectively, yielding *k*_*X*f_ =(7 ± 2) × 10^8^ s^–1^.

### Quantum-Chemical Modeling

DFT calculations were performed for **BMP** and its methyl-free analogue (**BBP**) and compared with the results obtained for **BBHQ**, which has two OH groups in the central ring (Scheme 2).

**Figure 8:**
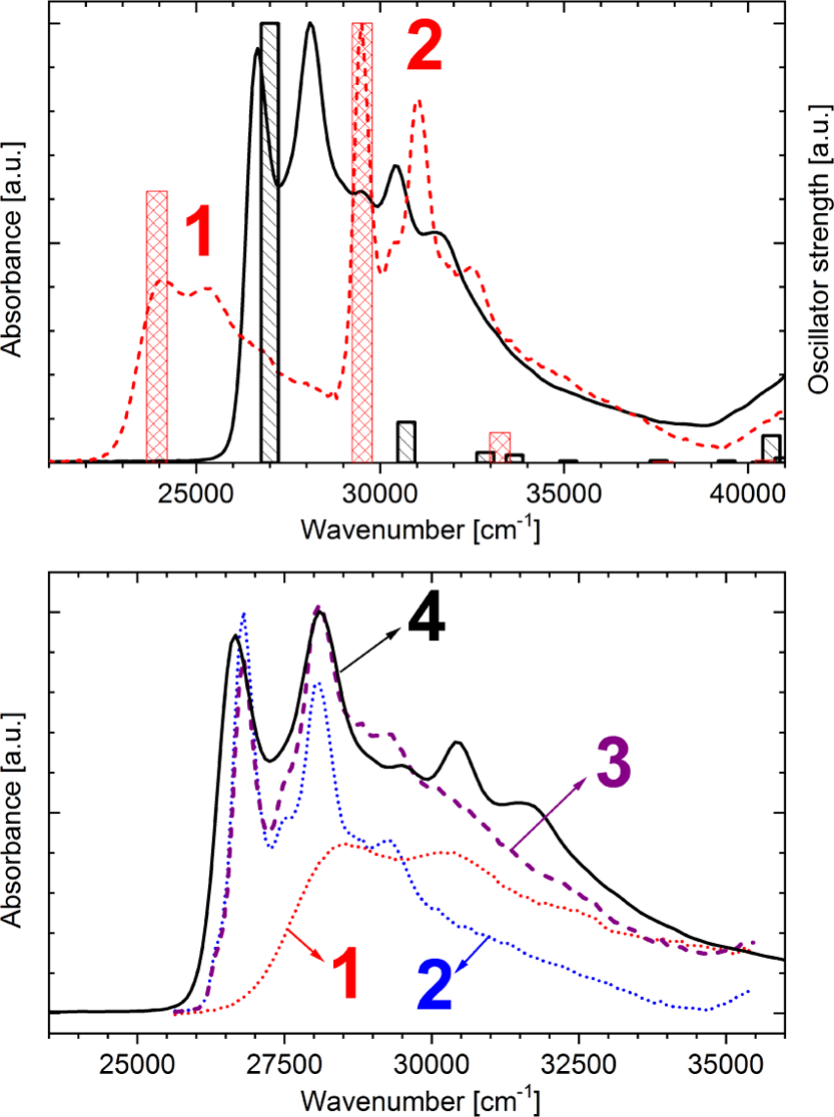
Top: room-temperature absorption spectra of **BMP** (black, solid) and **DE-BBHQ** (red, dashed) recorded in *n*-hexane. Black and red bars indicate the TD-B3LYP-calculated S_*n*_ ← S_0_ transitions. Bottom: reconstruction of the first absorption band of **BMP** (3) using the sum of high- and low-energy bands of **DE-BBHQ**, red-shifted by 4500 cm^–1^ (1) and blue-shifted by 3000 cm^–1^ (2), respectively. For comparison, the room-temperature absorption spectrum of **BMP** (4) is also shown.

To investigate the nature of the excited states of **BMP** involved in the ESIPT reaction, ground-state (B3LYP) and excited-state (TD-B3LYP, TD-CAM-B3LYP, TD-M11) quantum-chemical calculations were performed. The comparison of the recorded and calculated absorption spectra of **BMP** is given in Figure S6. The best agreement was obtained for the B3LYP functional. It seems reasonable to compare the absorption spectra of **DE-BBHQ** with the absorption spectrum of **BMP**. Within the spectral window of 20 000–40 000 cm^–1^, the absorption spectrum of **DE-BBHQ** consists of two well-separated bands, whereas for **BMP**, only one band is observed in this spectral region (Figure 8, top).^40^ Quantum-chemical calculations clearly show that the first absorption band of **BMP** consists of two, S_1_ ← S_0_ and S_2_ ← S_0_, close-lying transitions, whereas in the case of **BBHQ**, two low-lying transitions are well separated (Figure 8, top). The first absorption band of **BMP** can be acceptably reproduced by high- and low-energy bands of **DE-BBHQ** shifted appropriately (Figure 8, bottom).

**Figure 9:**
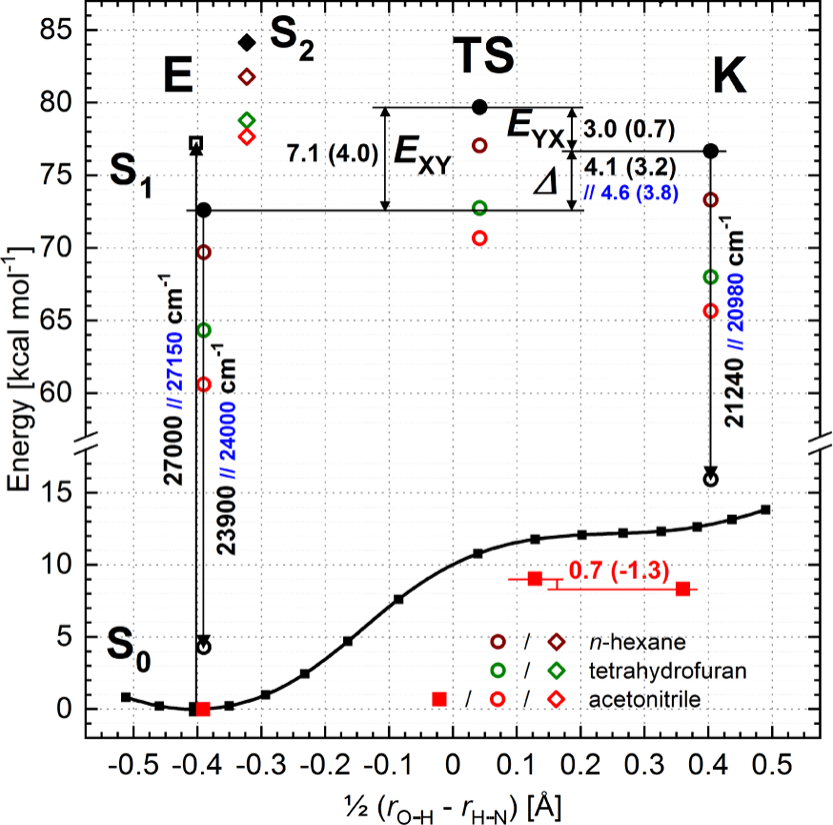
(TD-)B3LYP-calculated energy profile along the PT reaction path in the S_0_ state of **BMP** (line, squares) and the S_0_ and S_1_ energies of the enol (E) and keto (K) forms and the TS between them and S_2_ for the enol in vacuum (black; full symbols—optimized states, open symbols—vertically excited states), *n*-hexane (brown), THF (green), and acetonitrile (ACN) (red symbol) solutions (PCM solvation model; for the excited states, the vacuum-optimized geometries are used). Energy differences are given by numbers (in parentheses, after ZPVE correction). The relevant spectroscopic transitions are marked with arrows. The blue numbers indicate the results for rotamer II (Scheme 3).

According to the molecular modeling, the S_0_ energy profile of **BMP** in vacuum shows a single minimum, which corresponds to the enol form (Figure 9). In the region of the keto form, only a flattening of potential is observed, with the energy around 4400 cm^–1^ (12.5 kcal/mol) higher than that of the enol form. In contrast, in the S_1_ state, two minima of comparable depths corresponding to the enol and keto forms are easily localized. However, independent of the functional used (Table 3), the keto form has a higher energy (by 0.1–3.2 kcal/mol, see Figure 9, Table 3).

The effect of solvation on the PT reaction was checked using the PCM solvation model. It is usually elaborated on the basis of the Onsager model, in which the molecule is located in the Onsager cavity characterized by the radius *a*_0_, evaluated from the molecular dimensions.^49^ The solvent is approximated by a continuum, characterized by a polarity function *F*(ε,*n*), where ε is the relative permittivity and *n* is the refractive index and has nonzero values also for nonpolar solvents.^50,51,53^ An alternative model which also explains the nature of solvent stabilization in nonpolar media was proposed by Berg.^54^ From the plot of the solvatochromic shift of the fluorescence maximum versus polarity function *F*(ε,*n*),^51−53^ a parameter (μ_e_(μ_e_ – μ_g_)/(*a*_0_)^3^) can be evaluated, where μ_e_ and μ_G_ are the dipole moments of the S_1_ and S_0_ states, respectively. The B3LYP-calculated values of the dipole moment in the S_0_, S_1_, S_2_ states of the enol and the S_0_, S_1_ states of the keto form of **BMP** are 1.7, 1.7, 3.2, and 4.8, 6.8 D, respectively (Figure S8). The identity of the dipole moments in the first excited singlet and ground states explains why the solvatochromic shift is not observed for the emission originating from the S_1_ state of the enol form of **BMP**. In the case of the fluorescence from the S_1_ state of the keto form, the difference between fluorescence maxima in MTHF and 3MP is only 250 cm^–1^, which indicates that the values of the dipole moments of the S_1_ and S_0_ states of the keto form are also similar.

It should be stressed that the PCM formalism, in comparison with the classical Onsager model, provides a more realistic description of the molecular skeleton and, consequently, a more precise description of the solvent cavity and the exact electron density distribution of the molecule, rather than multipole expansion, is responsible for the continuum polarization. The PCM-calculated solvation energies of the S_0_ and S_1_ states of **BMP** and the enol form of the S_2_ state in three different solvents of increasing polarity (*n*-hexane, THF, and ACN) are presented in Figure 9 and Table 3. For the S_0_ state of **BMP** in ACN, not only a decrease of the keto–enol energy difference is predicted (as expected, based purely on the calculated dipole moments) but also the formation of a shallow energy minimum for the keto form is predicted (Figure 9). In contrast, in the S_1_ state, the keto form is only slightly more stabilized by solvents than the enol one (even a reversed tendency is observed for the B3LYP functional and the ACN solvent). One has to note that the S_2_ state of the enol form is substantially less stabilized than both the enol and keto forms in the S_1_ state.

In the end, it has to be mentioned that independent of the state and form, the conformation of the methyl groups in **BMP** was fixed to that as in the ground state of the enol form. This was not always optimal, but it was checked that their rotation did not change the energy of the system by more than 0.2 kcal/mol.

**Figure 10:**
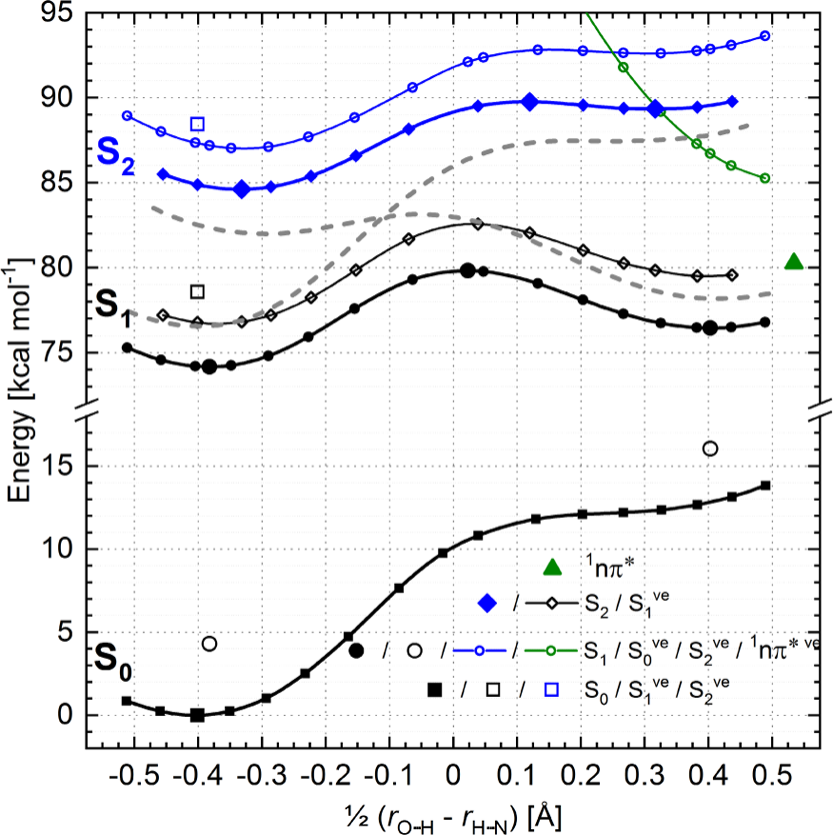
(TD-)B3LYP-calculated energy profiles along the PT reaction path for the S_0_, S_1_, S_2_, and ^1^*n*π* states of **BBP**. Squares (S_0_), circles (S_1_), diamonds (S_2_), and triangles (^1^*n*π*) indicate the state for which the geometry was optimized (full symbols). Dashed gray lines show the energy profiles of the hypothetical diabatic states, which upon interaction (a coupling term of 1551 cm^–1^) form the calculated S_1_ and S_2_ curves.

More detailed calculations were performed for the methyl-free analogue of the **BMP**: **BBP** molecule. The energy profiles, dipole moments, and oscillator strengths for both systems are almost the same (Figures 9, S7, S8; Tables 3, S1). For **BBP**, we have calculated the energy profiles along the PT coordinate for the S_0_, S_1_, and S_2_ states (Figure 10). The values of the dipole moment obtained for the S_0_, S_1_, S_2_ states of the enol and keto forms are 1.7, 2.1, 3.0 and 4.7, 6.4, 5.9 D, respectively. The orientation of dipole moments is almost the same as for **BMP** (Figure S8). The energy profiles calculated for the S_0_, S_1_, and S_2_ states of **BBHQ** are shown in Figure S9.

**Figure 11:**
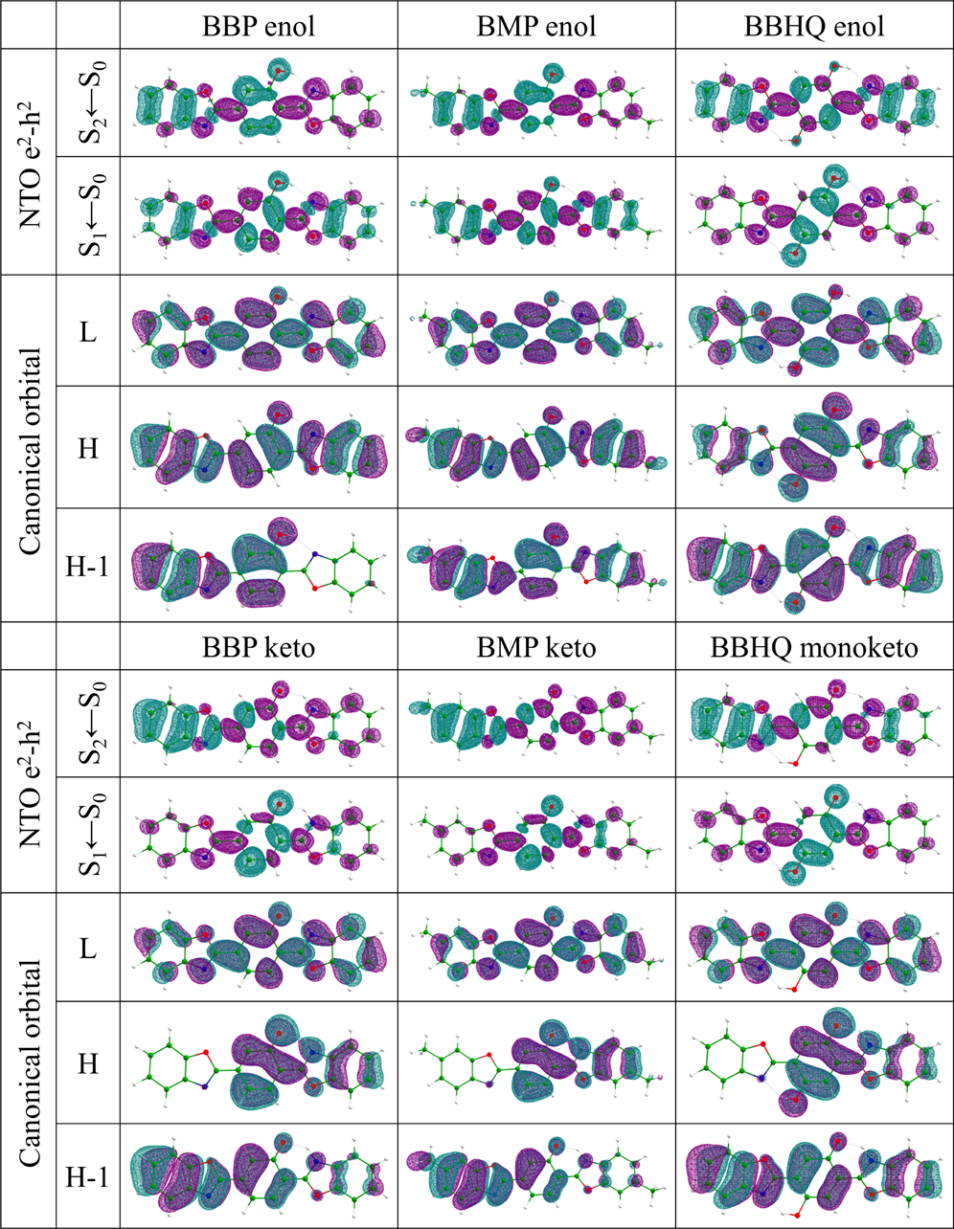
B3LYP-calculated shapes of molecular orbitals, HOMO – 1 (H – 1), HOMO (H), and LUMO (L) and differences of squares of NTOs (e—electron, h—hole) involved in S_1_ ← S_0_ and S_2_ ← S_0_ electronic transitions of enol (top) and keto (bottom) forms of **BBP**, **BMP**, and **BBHQ**. As a keto form, the geometry corresponding to the inflection point on the ground-state PT potential energy curve was taken.

The molecular orbitals involved in S_1_ ← S_0_ and S_2_ ← S_0_ electronic transitions of the enol and keto forms of **BBP**, **BMP**, and **BBHQ** are presented in Figure 11. For both forms, the lowest energy transition can be approximated by the HOMO–LUMO configuration, whereas the S_2_ ← S_0_ electronic transition can be approximated by the (HOMO – 1)–LUMO one. The LUMO orbital of the enol and keto forms of these molecules is similarly spread over the whole molecule. The same is true for the HOMO orbital of the enol form of **BBP** and **BMP**. Contrary to this, in the enol form of **BBHQ** and the keto form of all three systems studied, this orbital is mainly localized on the central (di)hydroxyphenyl part. Reversely, the HOMO – 1 orbital of **BBP** and **BMP** is localized on the “free” benzoxazole group and the central phenol ring, whereas in **BBHQ**, this orbital is spread over the whole molecule (Figure 11). The HOMO – 1 orbital of the keto form of all systems is localized on the “free” benzoxazole group and the central ring, however, without a significant electron density on the oxygen atom.

Differences in frontier orbital shapes correspond to changes in the partial atomic charges (Δ*q*), which occur upon excitation of the studied molecules. The electron density redistribution, mostly electron density flow from the central ring to the O–H···N-bonded benzoxazolyl group (HB-side), is the main driving force for ESIPT. Consequently, this parameter can be treated as a useful tool for predicting which excited state of the enol form has suitable properties for effective PT reaction. For **DE-BBHQ**, it was well established that upon excitation of the enol form to the S_1_ state, the monoketo form is generated very efficiently.^40^ Because the absorption and emission spectra of **DE-BBHQ** and **BBHQ** are almost identical, molecular modeling was performed for **BBHQ**. Indeed, calculations show that for the enol form of **BBHQ**, Δ*q* (central) is +303 me and Δ*q* (HB-side) is −151 me for the S_1_ ← S_0_ excitation. In contrast, upon excitation to the S_2_ state, the charge distribution change is much less pronounced (Table 4). Remarkably, for mono-OH substituted bis-benzoxazoles, the situation is reversed. A substantial charge redistribution favoring the PT is calculated for the S_2_ ← S_0_ electronic transition: Δ*q* (central) = +148, +147 m*e* and Δ*q* (HB-side) = −193, −205 m*e* for **BBP** and **BMP**, respectively, but not for the S_1_ ← S_0_ transition. In the S_1_ state of the keto form of **BBP** and **BMP**, the Δ*q* values are similar to those calculated for the keto form of **BBHQ**.

The hydrogen bond (HB) length (*d*_OH···N_), which correlates with the HB strength, is another very significant factor influencing the PT reaction dynamics. The calculations clearly show that *d*_OH···N_ in the S_1_ state of **BBHQ** (167 pm) is significantly smaller than that in S_2_ (179 pm), with the latter being similar to the ground-state value (181 pm). It indicates that upon excitation to the S_1_ state, the enol–keto transformation occurs more effectively than in the ground and S_2_ states. Again, the situation is reversed for the enol form of **BMP** and **BBP**. The *d*_OH···N_ in the S_2_ state has a considerably smaller value than in S_0_ and S_1_ states. The *d*_OH···N_ in the S_1_ state of the keto form of **BMP** and **BBP** is similar to that of **BBHQ**.

Yet another very sensitive parameter of the HB strength is the O–H stretching frequency (ν_OH_). From the 74 cm^–1^ blue shift of the (0,0) S_1_–S_0_ transition upon OH/OD exchange, a significant decrease of the ν_OH_ after the S_1_ ← S_0_ photoexcitation was estimated for the *t*-butyl analogue of **BMP**, from 3050 to 2455 cm^–1^.^41^ It should be even higher than that expected for **BBHQ** (57 cm^–1^ blue shift). Our modeling shows that a significant decrease of the ν_OH_ is indeed predicted for the S_1_ state of **BBHQ** (−548 cm^–1^, Table 4). However, for the enol form of **BMP** and **BBP**, the calculated change is small for the S_1_ state (−94 and −149 cm^–1^) but large for the S_2_ state (−513 and −423 cm^–1^). It again indicates that a substantial strengthening of the HB, similar to that predicted for the S_1_ state of **BBHQ**, occurs in the S_2_ state of **BMP**/**BBP** but not in their S_1_ state.

Summing up, the analysis of several quantum-chemical parameters shows that the ordering of the two lowest excited states in the enol form of **BMP** and **BBP** is inverted in comparison with **BBHQ**. While the S_1_ state of **BBHQ** has typical properties of a state for which the PT reaction is favored (let us call it S^PT^), in the case of **BMP** and **BBP**, such properties are displayed by the S_2_ state. Correspondingly, the S_2_ state of **BBHQ** and the S_1_ state of **BMP**/**BBP** can be described as weakly favoring or nonfavoring the PT reaction states (S^*n*PT^). On the other side, the properties of S_1_ and S_2_ states of the (mono)keto form of all three molecules studied are pretty similar. It is nicely visualized by plots of differences of squares of natural transition orbitals (NTOs) involved in S_1_ ← S_0_ and S_2_ ← S_0_ electronic transitions (Figure 11), which envisage electron density redistribution accompanying photoexcitation.

It seems reasonable to assume that in the case of **BMP**/**BBP**, the S_2_ state of the enol form corresponds to the S_1_ state of the keto form and, correspondingly, the S_1_ state of the enol form relates to the S_2_ state of the keto form. The energy profiles for these hypothetical diabatic states are marked by dashed lines in Figure 10. Their PT and non-PT characters are clear. The peculiar shape of the modeled adiabatic S_1_ and S_2_ curves results from the strong coupling (1551 cm^–1^) between postulated diabatic states. Consequently, the results of molecular modeling of **BBP**/**BMP** can be interpreted in terms of inversion of the two lowest excited singlet states, nonfavoring and favoring PT, occurring along the reaction path.

## Discussion

### Isolated **BMP**

We start by recalling the results obtained for **BMP** isolated in supersonic jets.^35,37^ The main findings are the following:

- The primary fluorescence is not detected under the supersonic jet conditions,
- ESIPT reaction is irreversible and occurs via a tunneling process,
- proton/deuteron-transfer rate constants are *k*_T_ = 1.4 × 10^13^/1.2 × 10^12^ s^–1^,
- Two ground-state rotamers generated by the rotation of the ″free″ benzoxazole group are detected.

Both rotamers of **BMP** display a high-intensity (0,0) band in their fluorescence excitation spectrum monitored at the keto fluorescence. The ESIPT kinetics critically depends on the excited vibration that brings closer atoms engaged in the formation of the HB.^35,40^ The vibrations 99/100 cm^–1^ (rotamer I/II) and 40 cm^–1^ (I and II) are assigned to in-plane bending, and another one, 264/262 cm^–1^ (I/II), is assigned to an in-plane stretching mode.

### **BMP** in Solutions

Contrary to the results obtained for jet-isolated **BMP**, the separation of two different rotamers was not possible for solutions. Room- and low-temperature ^1^H NMR spectra of **BMP** presented in Figure S1 exhibit one set of signals even at the lowest temperature. This means that either only one rotamer of **BMP** is present in the solution or there is a fast exchange between rotamers on the NMR time scale. The observed temperature shift of the NMR signals can be related to the changes of the O–H···N HB strength and solvent polarity. The quantum-chemical calculations predict the existence of two ground-state rotamers close in energy (0.3 kcal/mol in vacuum, decreasing with solvent polarity to 0.0 kcal/mol in ACN) and separated by a relatively low rotational barrier (6.8 kcal/mol in a vacuum). It is reasonable to conclude that the rotamerization process in **BMP** in solutions is too fast for NMR detection.

### Stationary Absorption and Emission

The absorption and fluorescence spectra were recorded within the temperature range of 10–295 K. The vibrational structure of the first absorption band and the dual fluorescence pattern of **BMP** are almost independent of solvent polarity and temperature. However, in a nonpolar environment, below 153 K, the absorption spectrum of **BMP** changes, exhibiting the rise of a new fluorescence band with a maximum of about 22 000 cm^–1^ (Figure S2). This effect depends on concentration and can be associated with ground-state aggregation. No symptoms of such a process were observed for **BMP** in polar solvents.

High- and low-energy emission bands of **BMP** correspond to the enol and the keto forms, respectively. High-energy fluorescence, unstructured in MTHF at room temperature, exhibits a well-defined structural pattern below 223 K (Figure 2). In rigid MTHF, phosphorescence is observed (Figure 2). Its vibrational structure, corresponding well to that of the high-energy fluorescence band, and its spectral position indicate that the blue fluorescence and the phosphorescence originate from states of the same character. This conclusion is supported by the fact that the decay time of the primary fluorescence is equal to the rise time of the T_*n*_ ← T_1_ absorption band (Figure 7, Table 2) and the result of the quantum-chemical calculations (Figure 6). The maximum of the T_*n*_ ← T_1_ absorption band is 21 100 cm^–1^. The calculated energy of the dominant T_*n*_ ← T_1_ transition of the primary form is 18 600 cm^–1^, whereas for the secondary form, two bands located at 16 100 and 24 000 cm^–1^ are predicted.

**Table 2:** Decay Times (τ_d_) of the Primary Form and the Rise Times (τ_R_) of the Secondary Form Evaluated from the TRF (Figure 5) and TA (Figure 7) Spectra of **BMP** in MTHF at 93 K^a^

	τ_d_/ps	τ_R_/ps
TRF	15 ± 3 (19 900 cm^–1^)	17 ± 4 (24 000 cm^–1^)
TA	19 ± 4 (18 000 cm^–1^)	16 ± 4 (21 100 cm^–1^)
	16 ± 3 (23 900 cm^–1^)	

a The numbers in parentheses indicate the position of the band maximum.

Quantum-chemical modeling shows that for the keto form of **BMP**, the ^1^*n*π* state (*n* orbital localized on the carbonyl oxygen atom) is localized around 1800 cm^–1^ higher in energy than the lowest S_1_ (^1^ππ*) state (Figure S10). Such arrangement of the excited states could generate fast intersystem crossing. Consequently, the lowest triplet state of the keto form should be effectively populated. A rough estimation based on the energy gap between the S_1_ and T_1_ states of the enol form indicates that phosphorescence of the keto form should be observed at around 12 000 cm^–1^. Unfortunately, this spectral region was inaccessible for us. On the enol side, the lowest ^1^*n*π* state is localized far above the lowest ^1^ππ* states (Figure 10).

### ESIPT Reaction Kinetics

The formulae that describe the decay of the primary form and the rise and decay of the secondary one for the excited-state reaction were published by Birks more than 4 decades ago.^48^ For a reversible reaction (*k*_*XYT*_ > *k*_*X*_, *k*_*YX*_ > *k*_*Y*_), the fluorescence decay of the primary form should be biexponential. The fast component of the decay can be approximated by (*k*_*XYT*_ + *k*_*YX*_)^−1^; since in our case, *k*_*XYT*_ ≫ *k*_*YX*_, the slow one can be approximated as (*k*_*Y*_ + *k*_*YX*_)^−1^. The secondary form then rises and decays with those time constants, respectively. At room temperature, a biexponential decay of the blue fluorescence was observed. However, due to the limited temporal resolution and time window of the TRF apparatus, the evaluation of short and long decay times, respectively, was impossible. For **BMP** in MTHF, the ESIPT reaction can be treated as irreversible at temperatures lower than 200 K. In such a case (*k*_*YX*_ ≪ *k*_*Y*_), the decay of the primary form should be monoexponential with the rate constant given by *k*_*X*_ + *k*_*XYT*_. The secondary form decays with the *k*_*Y*_ rate. For **BMP** in a nonpolar solvent, the reaction is reversible within the whole studied temperature range of 173–297 K (Figure 3). The decay times of high- and low-energy fluorescence measured for **BMP** in the nonpolar solvent at room temperature are equal, proving the equilibrium established in the excited state.^35,37^ Due to the ground-state aggregation, the irreversible reaction temperature region was experimentally inaccessible.

**Figure 12:**
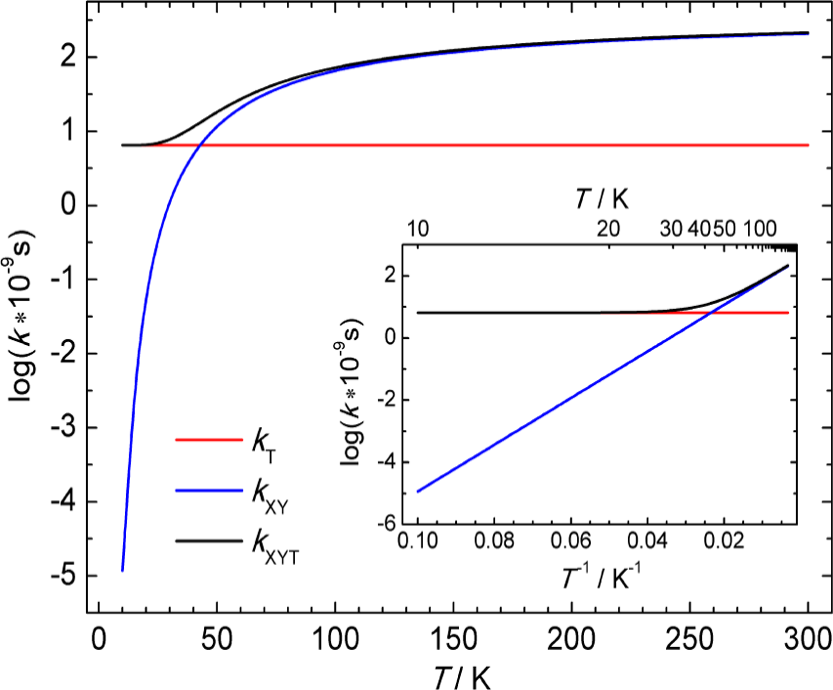
Temperature plot of the logarithm of *k*_*T*_, *k*_*XY*_, and *k*_*XYT*_ for reaction kinetic parameters evaluated from the experimental φ_*Y*_(*T*)/φ_*X*_(*T*) data for **BMP** in MTHF (*E*_*XY*_ = 120 cm^–1^, *A*_*XY*_ = 370 × 10^9^ s^–1^, *k*_*T*_ = 6.5 × 10^9^ s^–1^).

The decay/rise time of TRF and TA bands, assigned to the keto/enol forms, respectively, was evaluated at 93 K for **BMP** in MTHF (Table 2). A consistent value of 16 ± 3 ps was obtained, in perfect agreement with 15.0 ps, calculated from the evaluated kinetic parameters (Table 1). At *T* = 10 K, it should be equal to 125 ps, as approximated by (*k*_*X*_ + *k*_*T*_)^−1^ (Figure 12).

The Arrhenius energy barriers for enol → keto (*E*_*XY*_) and keto ← enol (*E*_*YX*_) reactions for **BMP** in MTHF and 3MP were determined from the fitting of φ_*Y*_(*T*)/φ_*X*_(*T*) and φ_*X*_(*T*) data sets (Figures 3, 4, Table 1). A relatively small value of 120 ± 30 cm^–1^ was obtained for *E*_*XY*_ in MTHF. It seems to be solvent polarity-independent. However, due to the limited temperature range available for **BMP** in nonpolar solvents, the value for 3MP is estimated with considerable uncertainty (Figure 3). As expected from the relative quantum yields of blue and red fluorescences, the back PT reaction barrier is substantially higher and depends on the solvent polarity (1600 ± 150 and 1200 ± 150 cm^–1^ in MTHF and 3MP, respectively).

The *k*_*T*_ value of 6.5 × 10^9^ s^–1^ was evaluated for **BMP** in MTHF. The *k*_*T*_ and *k*_*XY*_ rate constants become equal at around 40 K (Figure 12). Below this temperature, tunneling is the dominant channel of the reaction. The *k*_*X*f_/*k*_*Y*f_ ratio is 7, which indicates that the nature of the emitting state in the primary and secondary forms of **BMP** is different.

### Quantum-Chemical Modeling—A Critical Analysis

The DFT molecular modeling performed for the lowest excited S_1_ state of **BMP** incorrectly predicts the relative energy of the enol and keto forms (Figure 9, Table 3). Independent of the functional used, the ESIPT reaction in vacuum should occur uphill (0.1–2.3 kcal/mol) with a substantial energy barrier (1.7–4.0 kcal/mol, after ZPVE correction). It is inconsistent with the results of the experiments performed in supersonic jets and in the condensed media. This astonished us since similar calculations performed for **DE-BBHQ** and **BBHQ** predict almost identical energies of the enol and keto forms and a low energy barrier for tautomerization (0.6 kcal/mol), consistent with the experimental findings (Figure S9).^40^

**Table 3:** Relative Energies of Different Forms/Different States of **BMP** in Vacuum (in kcal/mol; in Parentheses, after ZPVE Correction) and Their Solvent Stabilization Energies Obtained by the PCM Model (See Figure 9), Calculated by Three Different Functionals

BMP	S_1_^enol^ – S_0_^enol^	S_1_^TS^ – S_1_^enol^ (*E*_*XY*_)	S_1_^keto^ – S_1_^enol^ (Δ)	S_2_^enol^ – S_1_^enol^
B3LYP				
vacuum	72.6 (70.3)	7.1 (4.0)	4.1 (3.2)	11.5 (11.0)
n-hexane	–2.9	–2.6	–3.4	–2.4
THF	–8.3	–6.9	–8.7	–5.4
ACN	–12.0	–9.0	–11.0	–6.5
CAM-B3LYP				
vacuum	81.8 (79.7)	5.9 (3.1)	2.5 (2.2)	13.8 (16.4)
n-hexane	–2.5	–2.3	–3.1	–2.1
THF	–6.2	–5.6	–7.3	–5.0
ACN	–7.7	–6.9	–8.9	–6.1
M11				
vacuum	87.0 (83.5)	4.2 (1.6)	0.2 (0.3)	14.5 (14.9)

Analysis of the properties of the modeled S_1_ state of the enol form (Figure 11, Table 4) shows that it does not have any characteristics of the state in which the PT reaction is favored (S^PT^) and can be denominated as an S^*n*PT^—a state nonfavoring the PT reaction. It somehow explains its uphill energy profile along the PT coordinate. However, the second excited state of the enol form, calculated to lie 3700 cm^–1^ above S_1_, has a strong S^PT^ character. Surprisingly, the PT potential energy curve for this state follows the S_1_ state uphill profile (Figures S9, S10). To explain this, we postulate that the remarkable shapes of the S_1_ and S_2_ state energy profiles along the PT coordinate in **BMP**/**BBP** are the result of the interaction between two diabatic states with the S^PT^ and S^*n*PT^ character, showing downhill and uphill energy profiles, respectively, and crossing each other along the reaction coordinate (Figure 10).

**Table 4:** Results of Quantum-Chemical Calculations (B3LYP) Performed for the S_0_, S_1_, and S_2_ States of the Enol and Keto Forms of **BBM**, **BMP**, and **BBHQ**: Δ*q*, the Change, upon Excitation, of the Electrostatic-Potential-Fitted Atomic Charges (in 10^–3^ of the Elementary Charge) Summed over the Selected Part of the Molecule (See Scheme 4); ν_OH_/ν_NH,_ the OH/NH Stretching Frequency; *d*_OH···N_/*d*_O···HN_, the Hydrogen Bond Length

molecule		BBP		BMP		BBHQ	
form		enol	keto^a^	enol	keto^a^	enol	mono-keto^a^
S_2_ ← S_0_ Δ*q* [me]	central	148	–86	147	–109	40	–5
	HB-side	–193	–203	–205	–213	–20	–146
	side	45	288	58	322	–20	152
S_1_ ← S_0_ Δ*q* [me]	central	87	203	40	196	303	267
	HB-side	–69	–86	–63	–83	–151	–84
	side	–17	–117	23	–113	–151	–183
ν_OH_/ν_NH_ [cm^–1^]	S_2_	2939	3023	2850	3065	3371/3323	b
	S_1_	3213	3286	3269	3287	2866/2859	3290
	S_0_	3362	3182	3363	3184	3414/3407	3054
*d*_OH···N_/*d*_O···HN_ [pm]	S_2_	168	168	166	170	179	b
	S_1_	176	184	177	184	167	183
	S_0_	179	176	179	176	181	170

a The keto form in the S_0_ state does not exist in vacuum. ν_NH_ and *d*_O···HN_ for that state are taken from the PCM modeling in ACN solution, and Δ*q* is calculated for the geometry corresponding to the inflection point on the PT potential energy curve for the S_0_ state.

b The keto form of BBHQ does not exist in the S_2_ state (see Figure S8).

This model is confirmed by the experimental findings showing that the first absorption band of **BMP** can be acceptably reproduced by the superposition of the two first well-separated absorption bands of **DE-BBHQ**: the unstructured S_1_ ← S_0_ and the structured S_2_ ← S_0_ (Figure 8, bottom). It has been well established that the PT reaction is promoted in the S_1_ state of **DE-BBHQ/BBHQ**.^40^ It is confirmed by DFT modeling, which additionally predicts that the S_2_ state of **BBHQ** is of the S^*n*PT^ type (Figure S9). For **BMP** in solutions, the structured component of the first absorption band lies somewhat lower in energy than the unstructured one. By analogy to **BBHQ**, it seems reasonable to conclude that the lowest excited state of **BMP** in solutions has the S^*n*PT^ character, but the S^PT^ state is closely located. On the other hand, it is well established that in vacuum, the lowest excited state of **BMP** has a strong S^PT^ character.^35,37^ Only the keto emission is observed, and the reaction rate is exceptionally high (*k*_*T*_ = 1.4 × 10^13^ s^–1^), more than 3 orders of magnitude higher than in solutions (6.5 × 10^9^ s^–1^ in MTHF).

### ESIPT Reaction Model

**Figure 13:**
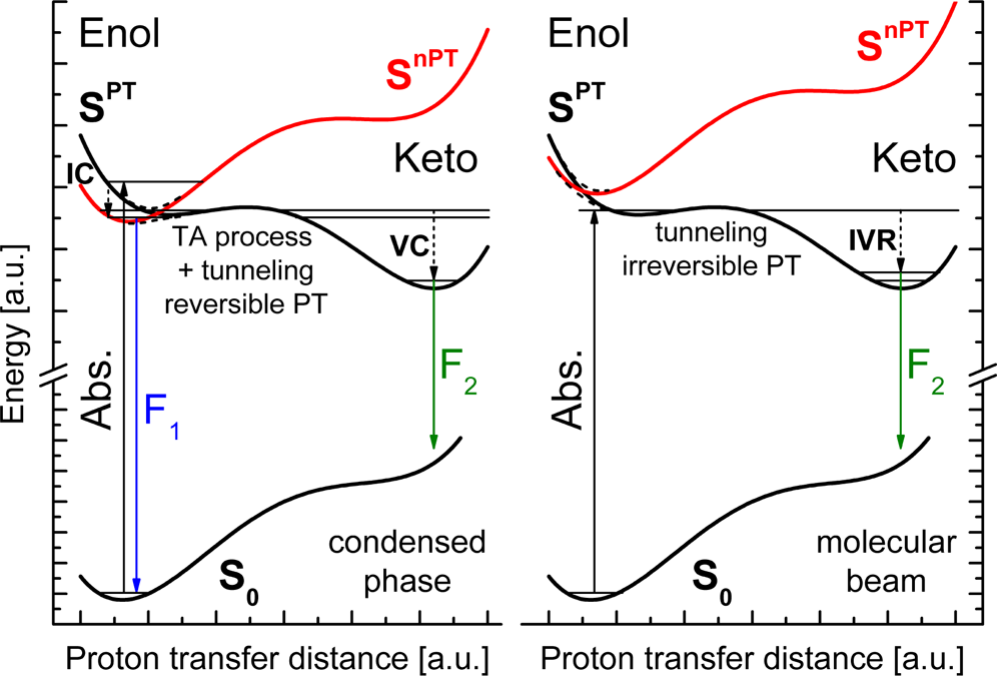
Proposed scheme of the energy levels of **BMP** in the condensed phase (left) and in vacuum (right). IC—internal conversion, VC—vibrational cooling, IVR—intramolecular vibrational redistribution, TA—thermally activated.

All the above considerations lead us to the model which consistently explains vacuum isolation and solution studies and, after some adjustment, the results of the quantum-chemical modeling. Let us shift down in energy by 4000 cm^–1^ the whole diabatic S^PT^ curve presented in Figure 10. This transformation locates the S^PT^ state lower in energy than the S^*n*PT^ one (Figure 13, right). Such ordering of the excited states explains the fast, almost barrierless ESIPT reaction observed for **BMP** in vacuum. Next, we assume that for **BMP** in solutions, the energy of the diabatic S^*n*PT^ state for the enol form is somewhat lower than that of S^PT^. As a consequence of the uphill and downhill potential energy profiles for the S^*n*PT^ and S^PT^ states, respectively, they cross at some point along the PT coordinate (Figure 13, left). In such a case, the resulting (non-)adiabatic lowest excited-state PT profile is characterized by a higher reaction barrier than in vacuum. Additional energy is required to reach the S^PT^ curve from the S^*n*PT^ minimum. It satisfactorily explains the difference in the ESIPT reaction kinetics for **BMP** in vacuum and solution.

According to the proposed model, the S^*n*PT^ state of the enol form of **BMP** should be more effectively stabilized by the dipolar environment than the S^PT^ one. Our DFT modeling using the PCM solvation formalism fully supports this (Figure 9, Table 3). Even in nonpolar *n*-hexane, the S_1_ (S^*n*PT^) state of the enol form of **BMP** is predicted to be 0.5 kcal/mol more stabilized than the S_2_ (S^PT^) state. Moreover, for our model system, **DE-BBHQ**, the blue solvatochromic shift of the first absorption band (S^PT^ ← S_0_) and the red shift of the second band (S^*n*PT^ ← S_0_) were observed and explained by DFT modeling.^40^ It provides additional strong support for our assumption. Based purely on the dipole moment values (6.8 and 1.7 D), one can expect that in the S_1_ state, the keto form should be more stabilized by the dipolar solvent than the enol one. It is consistent with the experimentally determined reaction enthalpy (*E*_*XY*_ – *E*_*YX*_), which is around 400 cm^–1^, more negative in MTHF than in 3MP. Our PCM modeling reproduces this behavior, but only when the CAM-B3LYP functional is used. It can be somehow rationalized, given the tendency of the B3LYP functional to overestimate charge-transfer character/dipole moments of some excited states and the strong mixing of the S^PT^ and S^*n*PT^ states. On the other hand, the calculated dipole moments of both tautomers are similar in the S_1_ and S_0_ states (Figure S8), explaining the lack of the solvatochromic shift of both fluorescence bands.

Finally, the main drawback of the DFT modeling has to be addressed. Assuming the correctness of our model, the energy of the S^PT^ state in mono-OH-substituted bis-benzoxazoles is calculated around 3500 cm^–1^, too high in comparison to the S^*n*PT^ one. It can be somehow rationalized by their substantially different properties (Figure 11, Table 4) and the well-known drawbacks of the TD-DFT method (e.g., wrong description of states with a charge-transfer character). We tried to address this issue by repeating our calculations using two range-separated functionals: a long-range corrected CAM-B3LYP and meta-GGA highly parameterized Minnesota M11 (Tables 3, S1). Indeed, the uphill shape of the ESPIT reaction profile for **BBP**/**BMP** systematically improves, but we are still far from the expected one as in Figure 13. Surprisingly, the S^PT^–S^*n*PT^ separation for the enol form does not decrease. However, the analysis of the nature of the S_1_ and S_2_ states of the enol form shows that upon going from B3LYP, through the CAM-B3LYP to M11 functional, their PT favoring character gradually exchanges. In the M11 case, both states have similar “average” PT properties (Table S2). It seems that further improvement of molecular modeling is possible. Probably, one has to go beyond the TD-DFT approach. Our preliminary ab initio calculation (CIS(D)) points to a potential double-excitation character of the S^PT^ state in **BMP**. Interestingly, this is not the case for doubly OH-substituted bis-benzoxazoles (Figure S9, Table S2). Further investigations, by means of spin-flip DFT or ADC(2) approaches, are planned in this field.

## Conclusions

The kinetics of the ESIPT reaction in **BMP** crucially depends on the energy ordering of the two lowest excited states in the enol form. In solutions, the ESIPT reaction is controlled by a thermally activated process and by the temperature-independent tunneling. The experimentally determined relatively small activation energy of 120 cm^–1^ can be interpreted in two ways: classically as the PT reaction potential energy barrier or alternatively, in terms of the vibrationally activated tunneling, as the frequency of the PT-promoting vibrational mode.^33^ Indeed, 120 cm^–1^ corresponds well with the frequency of the experimentally observed PT-promoting vibrational mode of 99 cm^–1^.^35,37^ At temperatures lower than 50 K, the temperature-independent tunneling plays a leading role. In vacuum, the tunneling and the intramolecular vibrational redistribution determine the extremely fast kinetics and irreversibility of the PT reaction. In vacuum, *k*_*T*_ is about 14 × 10^12^ s^–1^, but in condensed media, this value is only 6.5 × 10^9^ s^–1^. This is due to the inversion of the two lowest excited states occurring along the reaction path, which occurs in the condensed phase and generates an additional component to the ESIPT reaction barrier.

## Supporting Information Available

Atom numbering, stationary absorption and fluorescence, temperature dependence of relative fluorescence quantum yields, calculated relative energies, energy profiles, dipole moments, and transition energies (PDF)
