# Supplementary material for: Solving the Puzzle of Unusual Excited-State Proton Transfer in 2,5-Bis(6-methyl-2-benzoxazolyl)phenol
Source: J Phys Chem A. 2022 Mar 14;126(11):1823–36. doi: 10.1021/acs.jpca.1c10030 (PMC8958588; doi:10.1021/acs.jpca.1c10030)

Solving the Puzzle of Unusual Excited State Proton Transfer in 2,5-Bis(6-methyl-2-benzoxazolyl)phenol

Jacek Dobkowski,[1]* Michał Kijak,[1] Sylwester Gawinkowski,[1] Elena Karpiuk,[1†] Mariusz Pietrzak,[1] Igor V. Sazanovich,[1‡] Jacek Waluk[1,2]

[1] Institute of Physical Chemistry, Polish Academy of Science, Kasprzaka 44/52, 01-224 Warsaw, Poland.

[2] Faculty of Mathematics and Science, Cardinal Stefan Wyszyński University, Dewajtis 5, 01-815 Warsaw, Poland

[†] Present address: Institute of Physics, Polish Academy of Sciences, Al. Lotników 32/46, 02-668 Warsaw, Poland.

[‡] Present address: Central Laser Facility, Research Complex at Harwell, STFC Rutherford Appleton Laboratory, Harwell Science and Innovation Campus, Chilton, Oxfordshire, OX11 0QX, United Kingdom.

Supplementary material

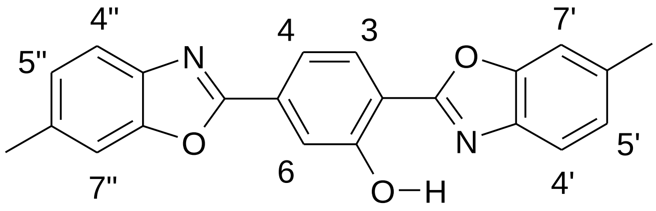

Scheme S1. Atom numbering in **BMP**.

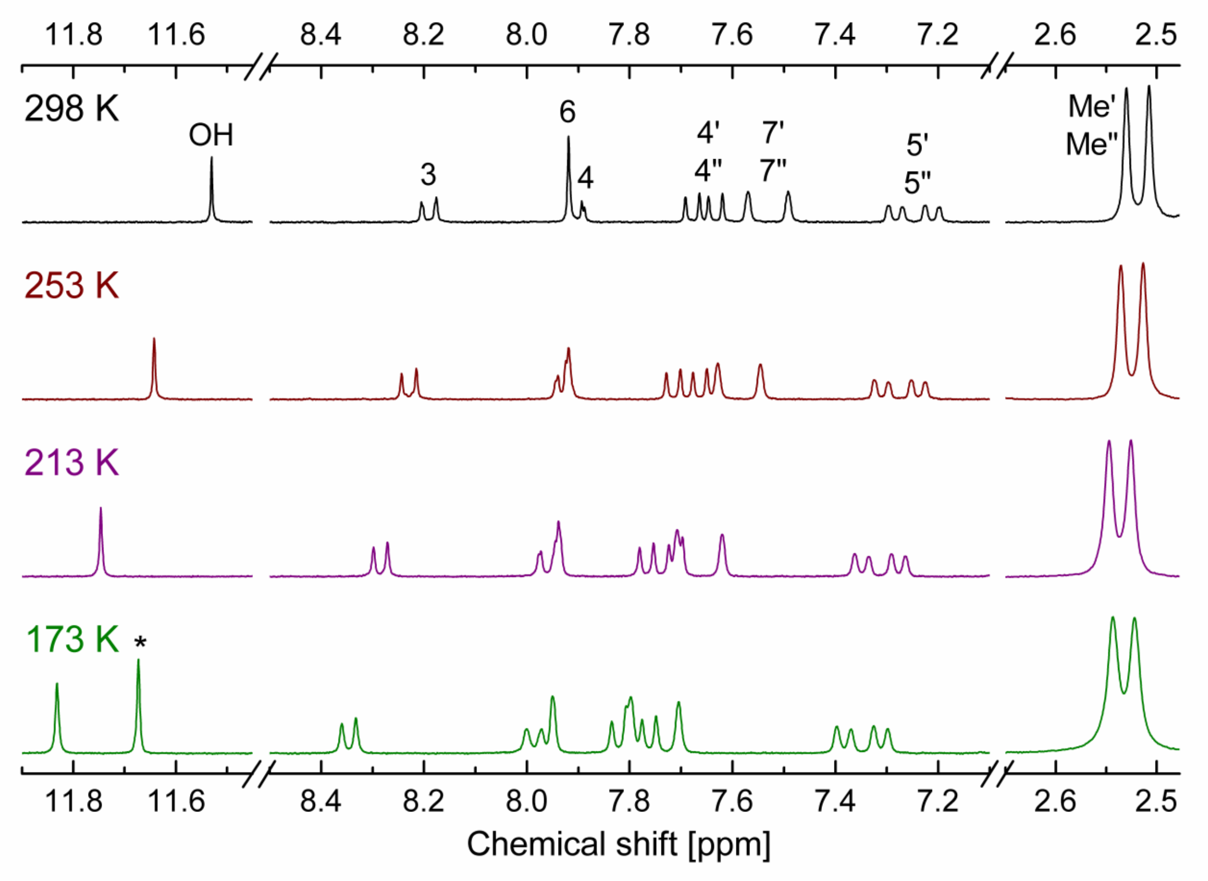

Figure S1. ^{1}H NMR spectra of **BMP** in THF-d_8 recorded at temperatures ranging from 298 K down to 173 K. * - solvent contamination.

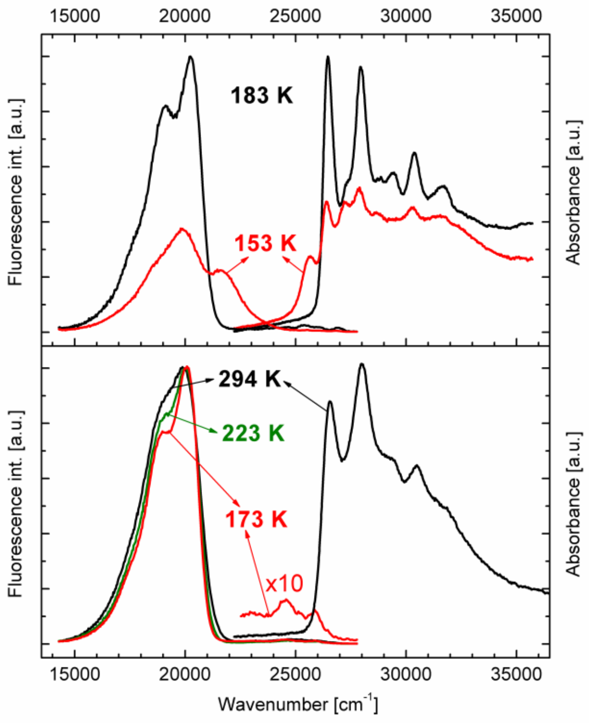

Figure S2. Stationary absorption and fluorescence (excitation at 28300 cm^{-1}) spectra of **BMP** in 3MP (top) and BuCN (bottom) recorded as a function of temperature.

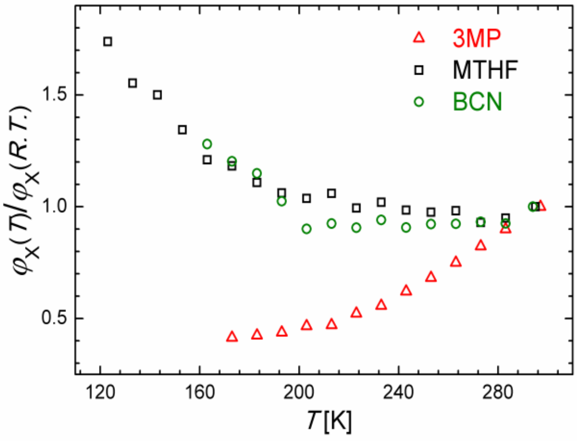

Figure S3. Temperature dependence of the relative quantum yield of the primary fluorescence of **BMP** in 3MP (red triangles), MTHF (black squares), and BuCN (green circles).

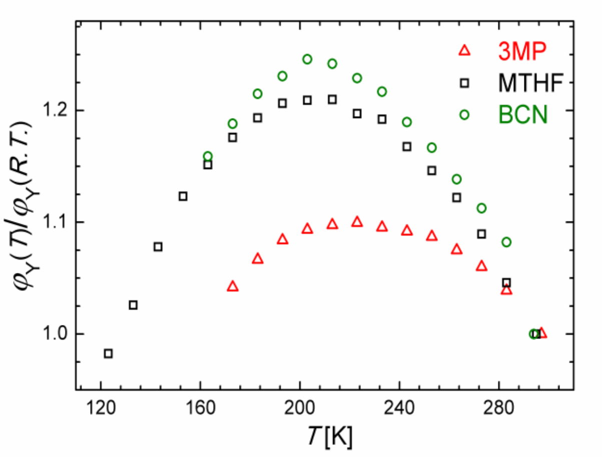

Figure S4. Temperature dependence of the relative quantum yield of the secondary fluorescence of **BMP** in 3MP (red triangles), MTHF (black squares), and BuCN (green circles).

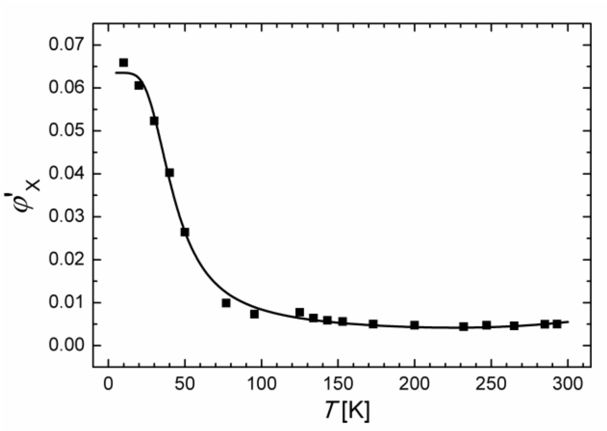

Figure S5. Temperature dependence of the raw quantum yield of the high energy fluorescence $\varphi'_X(T)$ of **BMP** in MTHF (squares) with the result of fitting with formula (1) (line, fitted parameters: E_{XY} = 107 ± 5 cm^{-1}, E_{YX} = 1500 ± 110 cm^{-1}, A_{XY} = (330 ± 50)·10^9 s^{-1}, k_X = (4.5± 0.2)·10^9 s^{-1}).

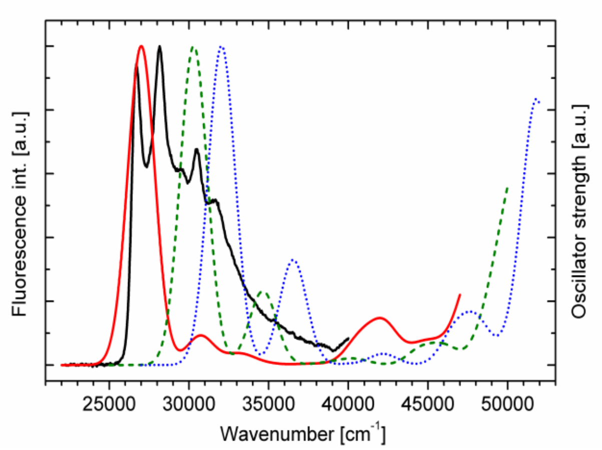

Figure S6. Comparison of the experimental absorption spectrum of **BMP** in 3MP (black) with the TD-DFT calculated one obtained using three different functionals: B3LYP (red solid line), CAM-B3LYP (green dashed line), and M11 (blue dotted line) and the S_0 B3LYP geometry.

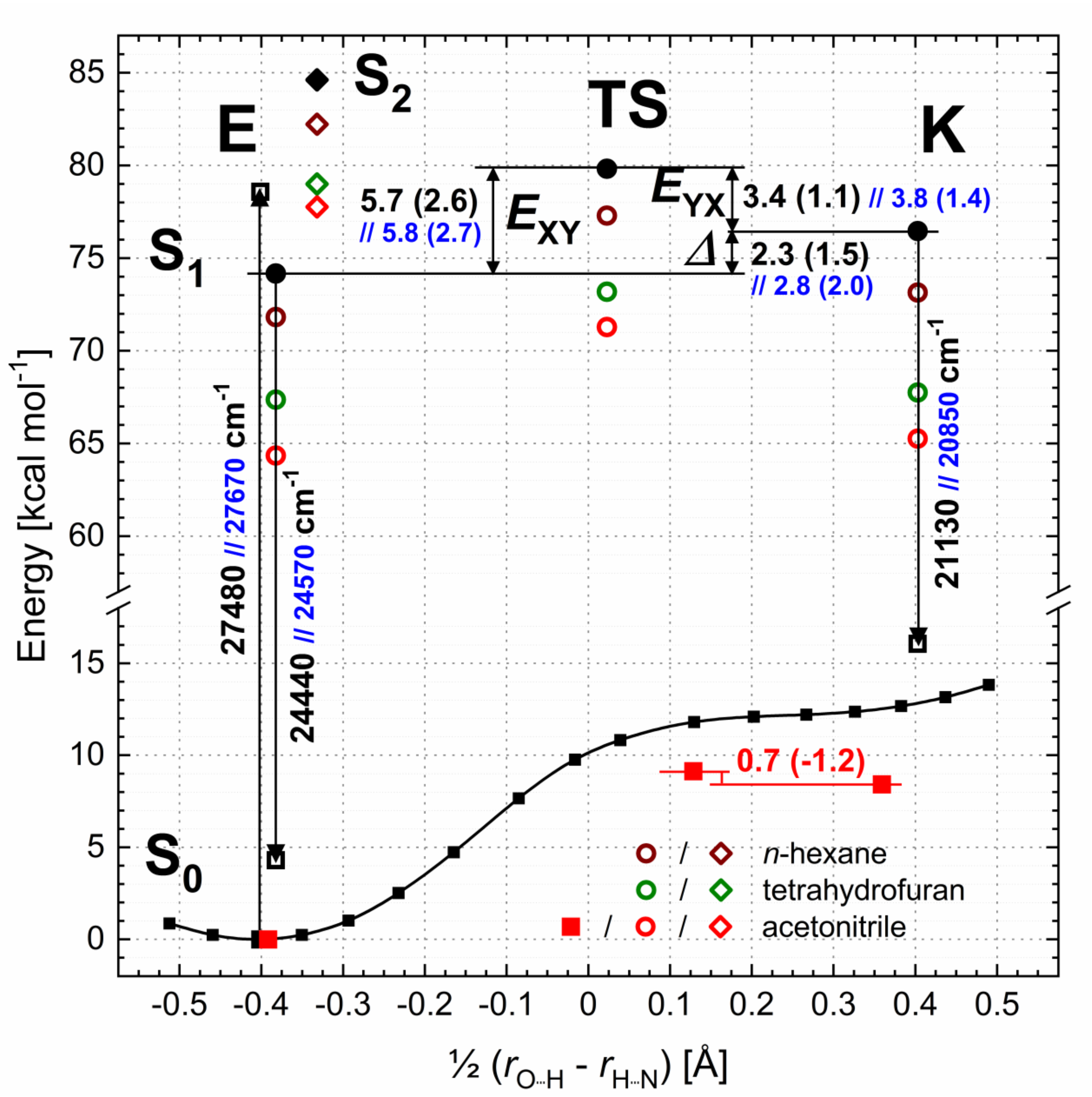

Figure S7. The (TD-)B3LYP calculated energy profile along the PT reaction path in the S_0 state of **BBP** (line, squares) and the S_0 and S_1 energies of the enol (E) and keto (K) forms, and the transition state (TS) between them, and S_2 for the enol in a vacuum (black; full symbols – optimized states, open symbols – vertically excited states), *n*-hexane (brown), THF (green), and ACN (red symbol) solutions (PCM solvation model; for the excited states the vacuum-optimized geometries are used). Energy differences are given by numbers (in parentheses, after ZPVE correction). The relevant spectroscopic transitions are marked with arrows. The blue numbers indicate the results for rotamer II (Scheme 3).

Table S1. The relative energies of different forms/different states of **BBP** in vacuum (in kcal/mol; in parentheses, after ZPVE correction) and their solvent stabilization energies obtained by the PCM model (see Fig. 9), calculated by three different functionals.

**BBP**	S_1^{enol} - S_0^{enol}	S_1^{TS} - S_1^{enol} (E_{XY})	S_1^{keto} - S_1^{enol} (Δ)	S_2^{enol} - S_1^{enol}
		B3LYP		
vacuum	74.2 (71.9)	5.7 (2.6)	2.3 (1.5)	10.4 (10.1)
n-hexane	-2.3	-2.5	-3.3	-2.4
THF	-6.8	-6.6	-8.7	-5.6
ACN	-9.8	-8.5	-11.2	-6.8
		CAM-B3LYP		
vacuum	82.9 (80.9)	4.9 (2.2)	1.3 (1.0)	13.0 (13.3)
n-hexane	-2.1	-2.2	-3.1	-2.1
THF	-5.1	-5.4	-7.2	-4.9
ACN	-6.3	-6.6	-8.8	-6.0
		M11		
vacuum	87.8 (84.7)	3.5 (1.0)	-0.6 (-0.6)	14.0 (14.5)

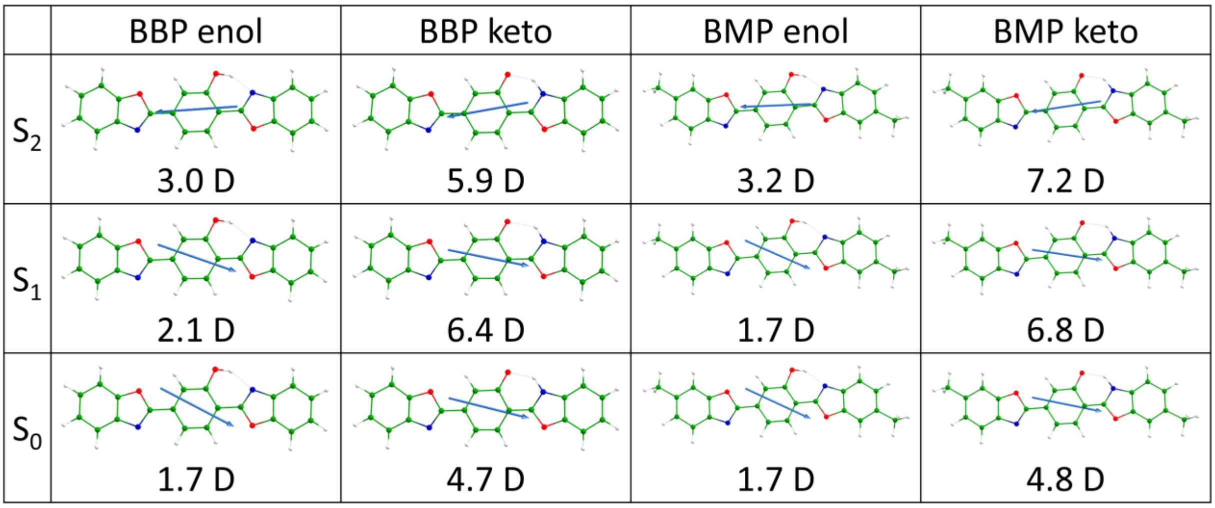

Figure S8. B3LYP calculated dipole moments of keto and enol forms of **BBP** and **BMP** in the S_0, S_1, and S_2 states. As a keto form in the ground state, the geometry corresponding to the inflection point on the potential curve was taken.

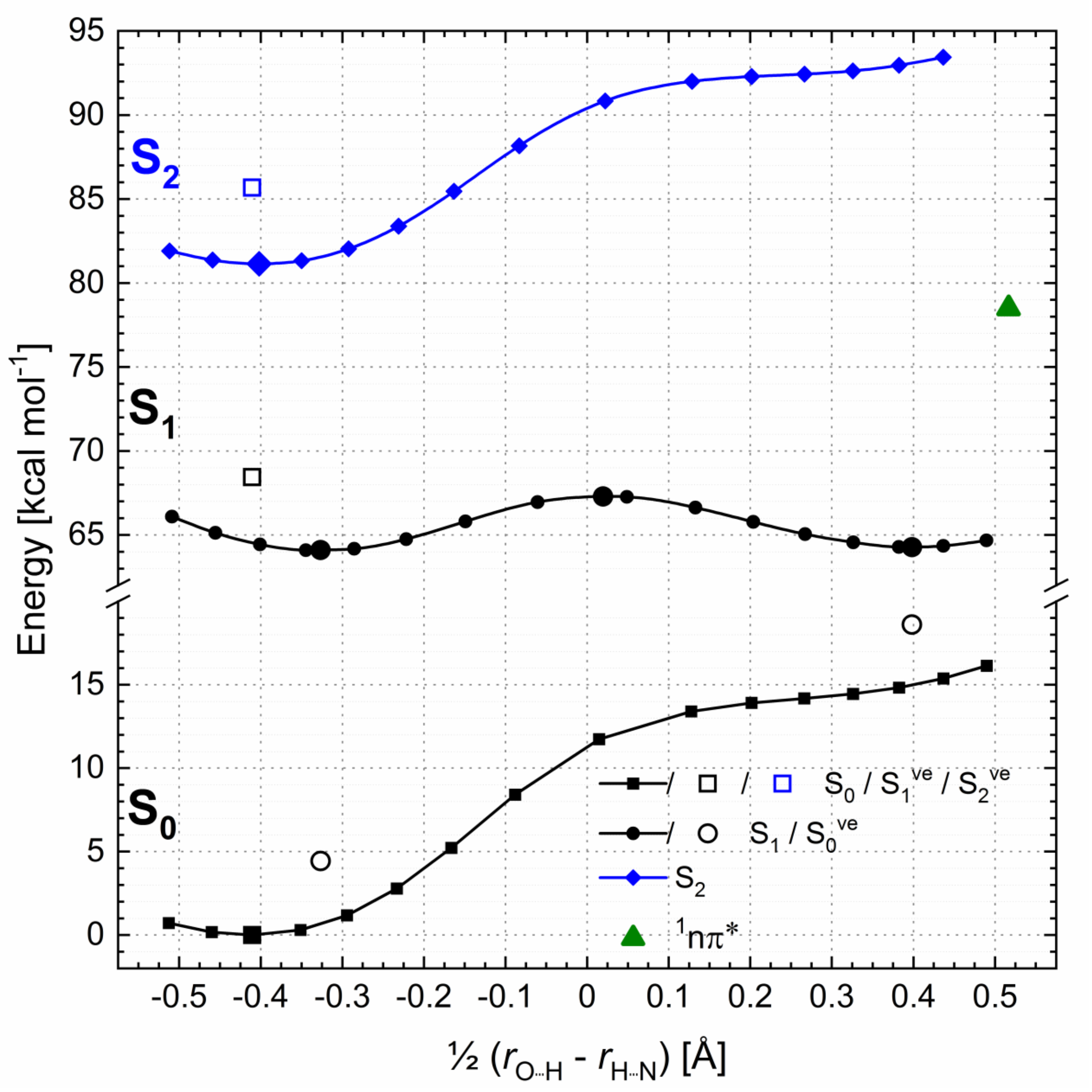

Figure S9. The (TD-)B3LYP calculated energy profiles along the PT reaction path for the S_0, S_1, and S_2 states of **BBHQ** (lines, full symbols). Vertically excited states (open symbols).

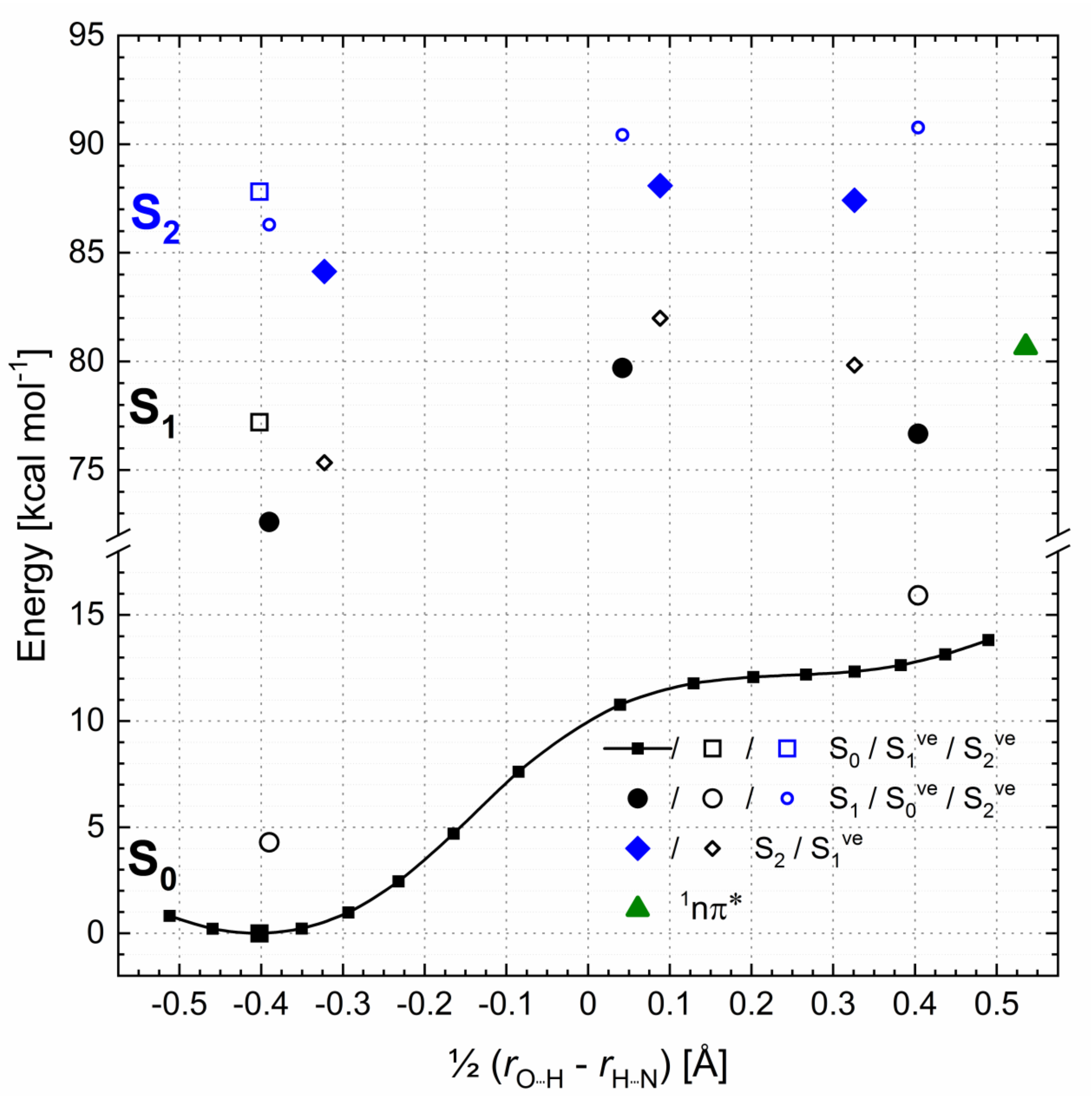

Figure S10. The (TD-)B3LYP calculated energy profile along the PT reaction path for the S_0 state of **BMP** (lines, squares) and the S_0, S_1, S_2 and $^1n\pi^*$ states energies of the enol (E) and keto (K) forms, and the transition state (TS) between them (full symbols – optimized states, open symbols – vertically excited states).

Table S2. The results of the quantum chemical calculations using B3LYP, CAM-B3LYP, and M11 functionals, performed for S_0, S_1, and S_2 states of the enol form of **BBM**, **BMP**, and **BBHQ**: Δq, the change upon excitation of the electrostatic-potential-fitted atomic charges (in 10^{-3} of the elementary charge) summed over selected part of the molecule (see scheme below); $\nu_{NH,}$ the NH stretching frequency; $d_{OH\cdots N}$, the hydrogen bond length.

Molecule		**BBP**			**BMP**			**BBHQ**		
Functional		B3L	CAM	M11	B3L	CAM	M11	B3L	CAM	M11
$S_2 \leftarrow S_0$ Δq [me]	central	148	148	137	147	150	136	40	96	113
	HB-side	-193	-120	-97	-205	-127	-102	-20	-48	-56
	side	45	-29	-39	58	-23	-35	-20	-48	-56
$S_1 \leftarrow S_0$ Δq [me]	central	87	141	172	40	108	147	303	278	273
	HB-side	-69	-98	-120	-63	-83	-107	-151	-139	-137
	side	-17	-43	-52	23	-25	-40	-151	-139	-137
ν_{NH} [cm^{-1}]	S_2	2939	3086	3152	2850	3022	3100	3371/ 3323		
	S_1	3213	3183	3035	3269	3248	3115	2866/ 2859	2917/ 2908	2916/ 2901
	S_0	3362			3363			3414/ 3407		
$d_{OH\cdots N}$ [pm]	S_2	168	169	173	166	168	171	179		
	S_1	176	173	171	177	174	173	167	166	167
	S_0	179			179			181		

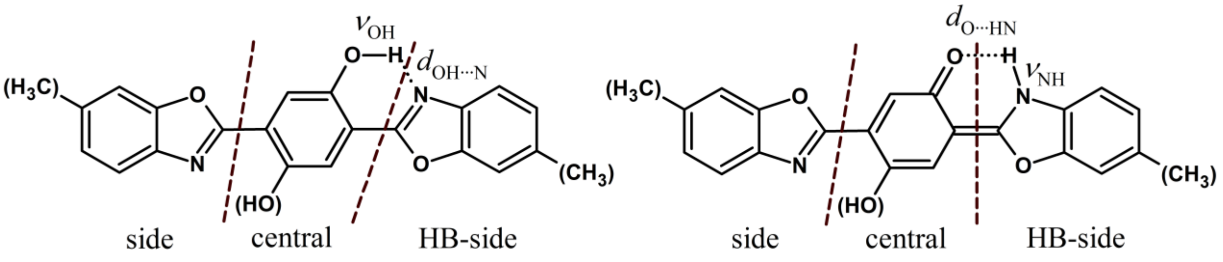

Supplement: Supplementary file 1 — jp1c10030_si_001.pdf [file jp1c10030_si_001.pdf]
